# Molecular basis of pigment structural diversity in echinoderms

**DOI:** 10.1016/j.isci.2024.110834

**Published:** 2024-08-30

**Authors:** Feng Li, Zhenjian Lin, Eric W. Schmidt

**Affiliations:** 1 Department of Medicinal Chemistry, University of Utah, Salt Lake City, UT 84112, USA

**Keywords:** Natural sciences, Chemistry, Inorganic chemistry, Chemical reaction, Catalysis

## Abstract

The varied pigments found in animals play both ecological and physiological roles. Virtually all echinoderms contain putative pigment biosynthetic enzymes, the polyketide synthases (PKSs). Among these, crinoids have complex pigments found both today and in ancient fossils. Here, we characterize a key pigment biosynthetic enzyme, CrPKS from the crinoid *Anneissia japonica*. We show that CrPKS produces 14-carbon aromatic pigment precursors. Despite making a compound previously found in fungi, the crinoid enzyme operates by different biochemical principles, helping to explain the diverse animal PKSs found throughout the metazoan (animal) kingdom. Unlike SpPks1 from sea urchins that had strict starter unit selectivity, CrPKS also incorporated starter units butyryl- or ethylmalonyl-CoA to synthesize a crinoid pigment precursor with a saturated side chain. By performing biochemical experiments, we show how changes in the echinoderm pigment biosynthetic enzymes unveil the vast variety of colors found in animals today.

## Highlights

• Complex crinoid pigments are made by an animal polyketide synthase
• A single mutation directs the pallet of pigment scaffolds in echinoderms
• Mutation conserved over hundreds of millions of years of echinoderm evolution

## Graphical abstract

**Figure:**
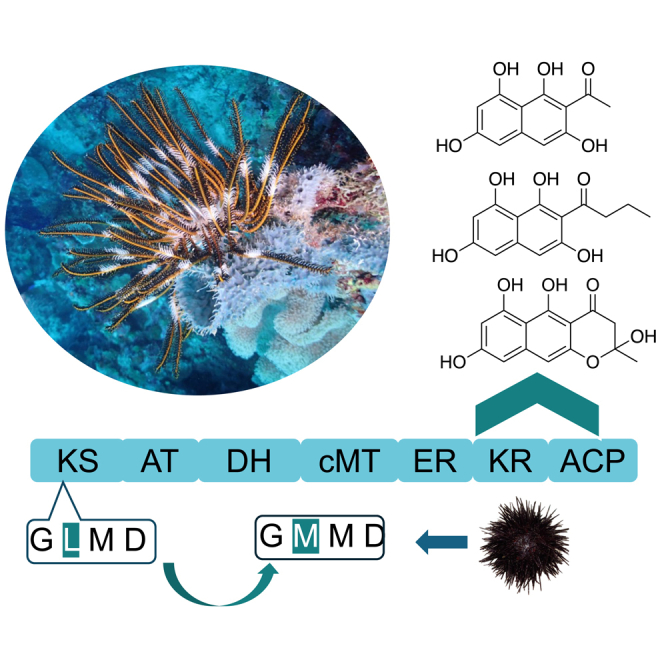

## Introduction

Since their early evolution, animals have maintained a biologically important and phylogenetically distinct set of polyketide synthase (PKS) enzymes, which in the few characterized examples produce compounds that are essential to animal survival.^1^^,^^2^^,^^3^^,^^4^^,^^5^^,^^6^ Biochemical studies show that the animal PKSs have distinct properties in comparison to those found in bacteria and fungi, which are much better characterized.^7^^,^^8^^,^^9^^,^^10^^,^^11^^,^^12^^,^^13^ Little is known about the chemical products of animal PKSs, and it remains impossible to predict the function of most of these enzymes despite their ubiquity across most animal groups.

**Figure 1:**
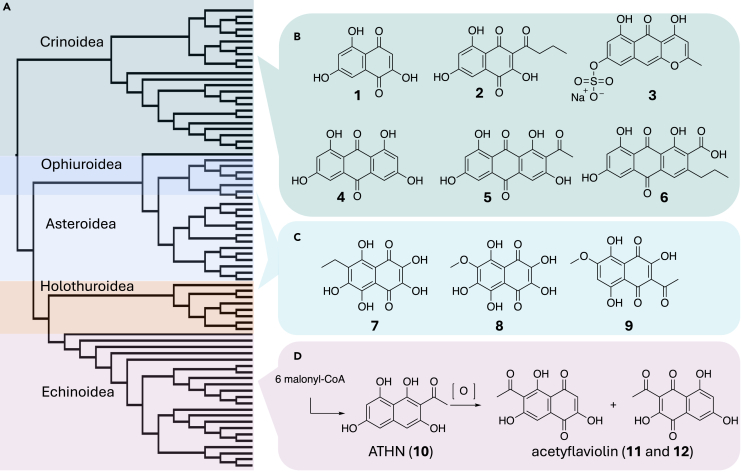
Polyketides and polyketide synthases (PKSs) from echinoderms (A) SpPks1 homologs found in echinoderms. The phylogenetic tree was made using ketosynthase (KS) domain sequence alignments and reflects the preservation of essential SpPks1 homologs over >500 million years of evolution. (B) Aromatic pigments previously reported from crinoids (Crinoidea) *Anneissia japonica*, which encodes CrPKS, and *Alloeocomatella polycladia*. (C) Examples of other echinoderm polyketide structural families from sea stars (Asteroidea), sea cucumbers (Holothuroidea), and brittle stars (Ophiuroidea). (D) Biosynthesis of ATHN (**10**) by the enzyme SpPks1 from sea urchins (Echinoidea). Further echinoderm-derived polyketides are shown in Figure S1.

Here, we sought to better understand the evolution and function of animal PKSs in a broadly occurring animal phylum, the echinoderms. Echinoderms are marine animals with 5-fold symmetry: the crinoids (Crinoidea), sea urchins (Echinoidea), sea cucumbers (Holothuroidea), brittle stars (Ophiuroidea) and sea stars (Asteroidea). They often contain PKS-derived, structurally diverse, colorful pigments comprised of aromatic polyketides, derived from naphthalene, anthraquinone, and napthopyrone carbon skeletons (Figures 1 and S1). These pigments are important to echinoderm survival, with both physiological and ecological roles.^14^^,^^15^ For that reason, the pigments have been reported widely from the living echinoderms^16^ and have even been isolated from fossil crinoids that are more than 200 million years old.^14^ The pigments have also been assayed in a wide variety of contexts, demonstrating biological activities such as antioxidant, antimicrobial, and cytotoxic effects.^14^^,^^15^^,^^16^^,^^17^^,^^18^^,^^19^^,^^20^^,^^21^^,^^22^^,^^23^^,^^24^^,^^25^^,^^26^ Thus, echinoderm pigments are ancient and central to animal survival.

Further reinforcing the central nature of echinoderm pigments, virtually every echinoderm genome encodes a PKS, one of which (SpPks1) was shown to produce a sea urchin pigment.^27^^,^^28^^,^^29^ SpPks1-like proteins occur in both the echinoderms and in their sister taxa, the acorn worms, indicating an ancient origin prior to the separation of these two phyla more than 500 million years ago (Figure 1A).^4^ This ancient origin reinforces biological studies demonstrating the importance of echinoderm pigments.^15^ The echinoderm PKS phylogenetic tree is congruent with both the pigment structural family and the echinoderm tree, indicating vertical transmission of the ancestral SpPks1 and conservation of function over evolutionary history (Figure 1A). The ancient echinoderm pigment structures are congruent with the phylogenetic tree (Figure 1B), with 12-carbon skeletons derived from one branch of the tree and larger skeletons derived from another, which diverged at least several hundred million years ago. Echinoderm PKSs are essential enzymes that have contributed to the wide success of echinoderms in modern oceans.

Echinoderm PKSs encode all needed biochemical domains as found in related enzymes, such as fungal highly reducing PKS (HRPKS) and animal type I fatty acid synthase (FAS), except that they lack the thioesterase (TE) domain involved in product release.^12^^,^^29^ The SpPks1 protein was biochemically characterized, synthesizing 2-acetyl-1,3,6,8-tetrahydroxynaphthalene (ATHN), the likely precursor of urchin pigments.^4^ The SpPks1 ketosynthase (KS), acyltransferase (AT), and acyl carrier protein (ACP) domains were necessary and sufficient for complete pigment biosynthesis, revealing a potentially unique route to aromatic polyketides.^4^ This implied that, potentially, the KS domain itself was responsible for folding and chain length determination. Since most of the variation in echinoderm pigments appears to result from the incorporation of different numbers of malonyl-CoA precursors (chain length), we hypothesized that different pigments would result from mutations in the KS active site.

Here, we tested this hypothesis by characterizing CrPKS from the crinoid *Anneissia japonica*, which contains the 14-carbon polyketide **2** and its likely metabolic product **1**, as well as longer polyketides **4**–**6**.^30^ CrPKS was expressed and purified, and the enzyme was used *in vitro* with malonyl- and acetyl-CoA substrates to demonstrate that it synthesizes 14-carbon crinoid pigment precursors, most of which incorporate an additional malonate unit in comparison to SpPks1 (Figure 1B). CrPKS also used butyryl-/ethylmalonyl-CoA to synthesize pigment precursors with saturated side chains. A structure-based approach identified key residues in the KS domain that, when mutagenized, modified the substrate tolerance and chain lengths produced by CrPKS. Recapitulating evolution, a single mutation in the KS domain of CrPKS made it function more similarly to SpPks1. These residues enable a domain-level understanding of chain length control and starter unit selectivity in the iterative PKSs from the >7,000 known species of echinoderms.

## Results

### CrPKS synthesizes crinoid pigment precursors

**Figure 2:**
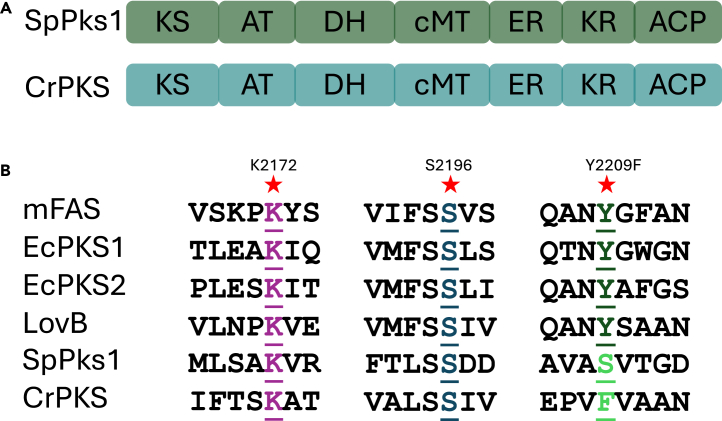
Architecture of CrPKS (A) CrPKS and SpPks1 have identical domain orders. Domains include: KS, ketosynthase; AT, acyltransferase; DH, dehydratase; cMT, a pseudo-methyltransferase structural domain with no catalytic activity; ER, enoylreductase; KR, ketoreductase; ACP, acyl carrier protein. (B) CrPKS KR domain is likely inactive, consistent with the synthesis of aromatic polyketides. Essential residue Y2209 is thought to be required for the activity of KR domains. It is present in a wide range of reducing PKSs and FASs from fungi and animals, but absent in the KR domain of SpPks1 and CrPKS, indicating a potentially inactive ketoreductase domain required for aromatic polyketide biosynthesis. Numbering according to CrPKS. mFAS: mammalian FAS, EcPKS1 and EcPKS2: biochemically characterized HRPKSs from mollusk *Elysia chlorotica*; LovB: HRPKS from fungus *Aspergillus terreus*.

**Figure 3:**
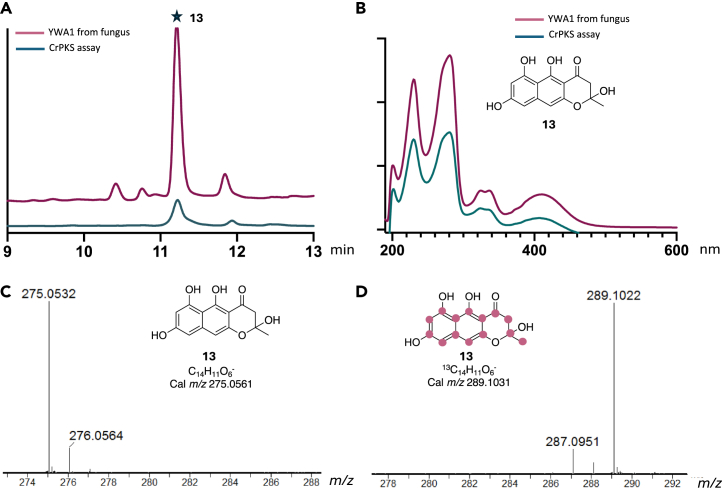
CrPKS synthesizes 14-carbon pigment **13** (A) HPLC (254 nm), extracted chromatograms. (B) UV chromatograms showing fungal products (magenta) and CrPKS reaction products (blue). (C and D) Mass spectra (negative mode) of **13** when CrPKS was incubated with (C) malonyl-CoA or (D) ^13^C malonyl-CoA. Purple dots indicate the incorporation of ^13^C. Purified protein is shown in Figure S2, while additional related data are shown in Figures S3–S6. Data showing the MS- and NMR-based identification of compound **13** can be found in Figures S7–S10. Figures S11 and S12 show that NADPH is not required and that either acetyl- or malonyl-CoA can be used as starter units.

The *A. japonica* crinoid PKS gene was assembled from SRA data available at NCBI (GenBank: SRR9663012).^31^ The resulting translated protein CrPKS was 76% similar to SpPks1. CrPKS and SpPks1 have the same domain architectures predicted by antiSMASH (Figure 2A), and both feature an inactive ketoreductase (KR) domain associated with aromatic polyketide biosynthesis (Figure 2B).^32^^,^^33^ Therefore, we proposed that CrPKS would synthesize aromatic compounds found in crinoids. The inactive KR domain is important because aromatic PKSs from fungi and bacteria commonly lack a KR domain entirely, consistent with their aromatic structures.^7^^,^^34^ CrPKS was expressed in *Saccharomyces cerevisiae* BJ5464 harboring the *npgA* phosphopantetheinyl transferase gene.^35^ Purified CrPKS (Figures 3 and S2) was incubated with malonyl-CoA leading to new peaks observed by UPLC-MS in negative mode at *m/z* 275.0586 (C_14_H_12_O_6_) and 289.0378 (C_14_H_10_O_7_) (Figures 3 and S3–S5). Incubation with [^13^C_3_]-malonyl-CoA led to peaks at *m/z* 289.1036, indicating that CrPKS incorporates 7 units of malonate (Figures 3 and S6).

The scaled-up enzyme reaction produced a green oil with a unique UV spectrum (λ_max_ 408, 336, 325, 280, 231, and 200 nm in CH_3_CN/H_2_O). In surveying related, 7-malonate aromatic polyketides, these data were most similar to those for YWA1 (**13**), an aromatic compound synthesized by fungal nonreducing PKS (NRPKS)^36^ corresponding to the MS peak at *m/z* 275.0586 (Figure S7). We isolated and purified YWA1 (**13**) from the fungus *Aspergillus nidulans* A1145+PKS47G. Dehydration of **13** to produce YWA2 (**14**), followed by NMR experiments, confirmed the compound identity (Figures S8 and S9).^37^ Co-injection of the confirmed standards with the CrPKS reaction products revealed that the enzymatic product was identical to YWA1 (**13**) based upon UV, retention time, and MS data (Figures 3 and S7) The higher molecular weight peak at *m/z* 289.1089 is likely to be the known YWA1 (**13**) oxidation product **16**,^34^ which was found in both the CrPKS enzyme product and in the *A. nidulans* extract (Figure S10).

As previously found with SpPks1, NADPH had no effect on the CrPKS reaction, with no new products observed by MS and no change in relative ratios of products formed (Figure S11). When ^13^C_1_-acetyl-CoA was included in the enzymatic reaction, **13** was observed with a 1 Da greater *m/z*, indicating that both acetyl-CoA and malonyl-CoA are accepted as CrPKS starter units (Figures S6 and S12).

YWA1 dehydration product **14** has the same carbon skeleton as **3**, a pigment isolated from the crinoid *Alloeocomatella polycladia*, which is in the same taxonomic family as *A. japonica* (Figure 1B).^38^ Moreover, **13** and related compounds are plausible biochemical precursors for *A. japonica* polyketides **2** and **1**.^30^^,^^39^ Because a small amount of the 12-carbon ATHN (**10**) was observed in enzyme assays, we also considered the possibility that *A. japonica* pigment **2** might instead originate from loading with a butyrate starter unit, rather than the later reduction of the side-chain carbon.

**Figure 4:**
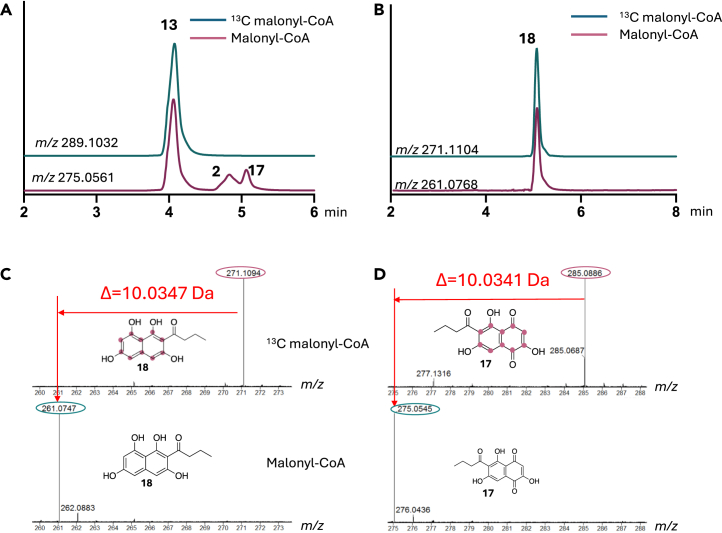
CrPKS synthesizes pigments with saturated side chains (A–D) Mass spectral chromatograms (negative mode) of the reaction mixture containing CrPKS and both malonyl- and butyrl-CoA, filtered for (A) *m/z* 275.0651 corresponding to compound **17** and (B) *m/z* 261.0768 corresponding to **18**. Enzymatic reactions were also performed with ^13^C-malonate, and the labeled (top) and unlabeled (bottom) mass spectra were compared for (C) compound **17** and (D) compound **18**. The addition of 5 units of malonate is reflected in the mass shift and further reinforced in Figures S13 and S4. Purple dots indicate the incorporation of ^13^C. Further MS data supporting this figure can be found in Figures S13–S17.

To test this hypothesis, we performed experiments with various alternative starter units. When butyryl-CoA was added to the reaction mixture, two minor products were detected at *m/z* 275.0548 and 275.0545 (*m/z* 285.0887 and 285.0886 with ^13^C_3_-malonyl-CoA), consistent with compounds **2** and **17** (Figures 4 and S13–S15). Compounds **2** and **17** are oxidized products of **18**, which were also detected at *m/z* 261.0747 (malonyl-CoA) and 271.1094 (^13^C_3_-malonyl-CoA), respectively (Figures 4C and S4). However, ethylmalonyl-CoA was a poor substrate (10% reaction products in comparison to butyryl-CoA), likely due to a limitation in starter unit decarboxylation (Figures S16 and S17).

In summary, CrPKS synthesized plausible biochemical precursors for previously reported *A. japonica* products **1** and **3**, in addition to compound **2** previously identified in a related crinoid.

### Ketosynthase domain controls chain length and starter unit selection

**Figure 5:**
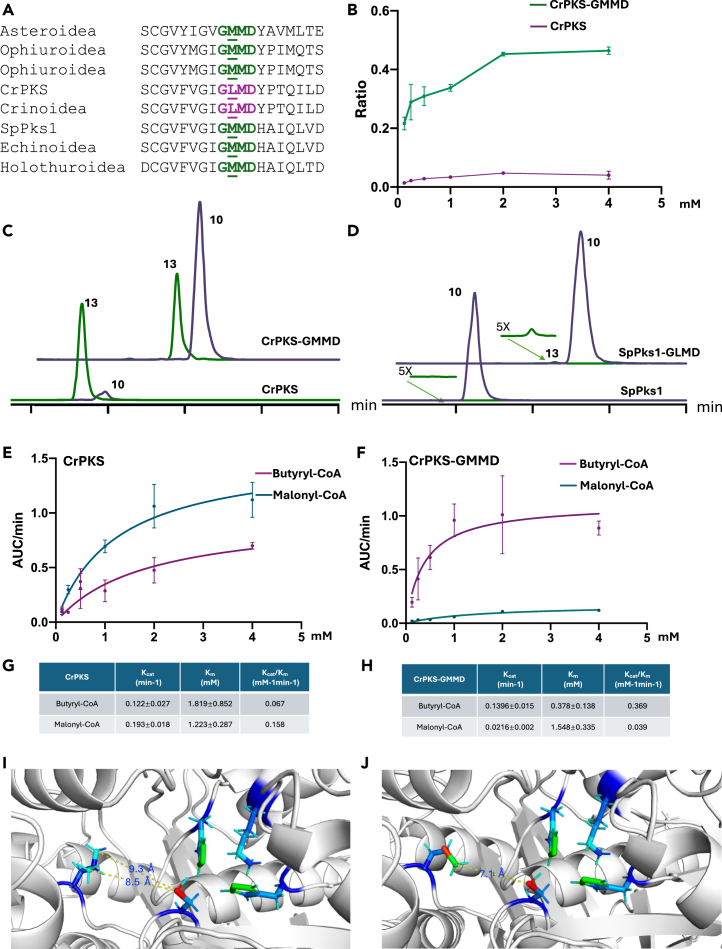
A KS domain mutation that controls chain length and starter unit selectivity (A) Conserved residues in SpPks1 and CrPKS. (B) Purified CrPKS-GMMD synthesizes more compound **10** than does wild-type CrPKS. The y axis is the ratio of [12-carbon products]/([12-carbon products] + [14-carbon products]) for CrPKS (magenta) and CrPKS-GMMD (green), while the x axis indicates the concentration of malonyl-CoA in mM. See Figures S19–S21 for details. Each point was replicated three times, with error bars representing the standard deviation. (C) In yeast cell pellets, CrPKS-GMMD synthesizes more compound **10** than does CrPKS (see Figures S23 and S24 for additional details). (D) In yeast cell pellets, SpPks1-GLMD synthesizes a small amount of compound **13** (see Figure S22). (E and F) Michaelis-Menten kinetics for CrPKS and (F) for CrPKS-GMMD. Curves are shown for different concentrations of malonyl-CoA (blue) or butyryl-CoA (magenta) in the presence of 2 mM malonyl-CoA. Each point was replicated three times, with error bars indicating standard deviation. (G an H) The corresponding kinetic constants generated from the curves shown in panels (E) and (F). Time course experiments underlying kinetics are in Figure S25. (I and J) AlphaFold models of the KS domains of (I) CrPKS and (J) SpPks1. The models show the distance between active-site cysteine and the mutated residue. Further models are shown in Figure S18. Primers used to generate mutants are found in Table S1.

SpPks1 produced only 12-carbon products, while CrPKS produced mainly 14-carbon products, with a minor amount of 12-carbon products (Figures 5C and 5D). We sought to determine the molecular basis of chain length control. In our previous study analyzing all known KS domains from every sequenced echinoderm, we noted conserved residues GM(L)MD adjacent to the active site cysteine in KS domains (Figures 5 and S18).^4^ KS domains are sometimes thought to impact the chain length of aromatic polyketides,^38^ and in our previous study of SpPks1, the KS was implicated in chain length determination.^4^^,^^40^ Out of 95 sequenced species of echinoderms, the G**M**MD sequence was observed in all genomes from sea urchins (45 specimens), sea stars (21 specimens), sea cucumbers (8 specimens), and brittle stars (3 specimens). The pigments in these echinoderm groups are 10–12 carbons in length. By contrast, the crinoid pigments are dominated by compounds with 14 or more carbons, and 17 out of 18 sequenced crinoid genomes contain the G**L**MD motif. No other mutations in the KS domains had such a clear pattern. Given the separation of Crinoidea and the remaining echinoderm taxa hundreds of millions of years ago, this is highly likely to be an evolutionarily significant mutation.^41^^,^^42^

Therefore, using AlphaFold,^43^ we predicted the structures of SpPks1 and CrPKS KS domains. In the resulting models, conserved residues GM(L)MD were close to the entry point of the KS active-site pocket (Figures 5I and 5J). The distance between the variable Leu/Met and the catalytic Cys residues was compared, indicating the potential that the space between Leu-Cys might be 1–2 Å longer than the Met-Cys space. We hypothesized that space in the KS active-site cavity dictates chain length.

Mutations SpPks1 M133L (SpPks1-GLMD) and CrPKS L130M (CrPKS-GMMD) were synthesized (Figure 5A and Table S1). Expressed CrPKS-GMMD was soluble and was used in pure form with various substrates in comparison to wild-type enzymes (Figures 5B, 5E, 5F, and S19–S21). Purified CrPKS and CrPKS-GMMD were incubated with different concentrations of malonyl-CoA, and the ratio of YWA1 (**13**) to ATHN (**10**) was calculated. With CrPKS, **10** was always a minor product comprising 1%–5% of all products (Figure 5B). In CrPKS-GMMD, **10** was present at 20%–50%.

SpPks1-GLMD was largely insoluble when expressed in *S. cerevisiae*. Fortunately, there was still some active protein in yeast cells, enabling experiments with yeast cell pellets in which the four mutant and wild-type proteins were compared (Figures 5 and S22–S24). In yeast pellet experiments, CrPKS-GMMD produced a higher ratio of 12-carbon products than those found in the wild type (Figures 5C, S22, and S23). In contrast to wild-type SpPks1 in which only 12-carbon products were detected, 14-carbon YWA1 (**13**) was detected in SpPks1-GLMD (Figures 5D and S24). These results support the importance of the KS in chain length control.

Purified CrPKS-GMMD was incubated with butyryl-CoA in the presence of malonyl-CoA (Figure S20). Compound **18** as well as oxidation products **2** and **17** were present in greater abundance than in wild type CrPKS, showing the starter unit preference seemed to be changed (Figure S21). Michaelis-Menten kinetics were performed for both CrPKS and CrPKS-GMMD with malonyl-CoA and butyryl-CoA (Figures 5E, 5F, and S25). In the presence of malonyl-CoA as substrate, the *k*_*cat*_ of CrPKS-GMMD was only 10% that of CrPKS. By contrast, in the presence of butyryl-CoA and malonyl-CoA, the *k*_*cat*_ of both enzymes was similar, but the catalytic efficiency for butyryl-CoA was significantly different. CrPKS had a *K*_*cat*_*/K*_*m*_ of 0.067 mM ^−1^min^−1^ for butyryl-CoA, while that of CRPKS-GMMD was 0.369 mM ^−1^min^−1^. The latter is even higher than the catalytic efficiency of the wild-type enzyme with solely malonyl-CoA (Figures 5G and 5H). This indicated that the KS mutation altered starter-unit selectivity, as well as chain lengths of products.

### Biosynthetic hypothesis

**Figure 6:**
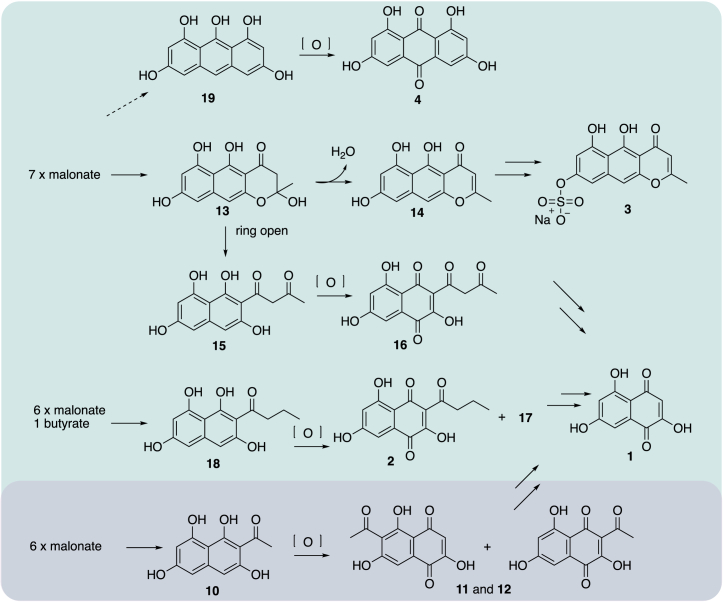
Plausible biosynthetic routes to pigments isolated from Crinoidea The top box indicates potential pathways surmised based on experiments with CrPKS, while the bottom box focuses on reactions catalyzed by SpPks1. Also, note that **13** is spontaneously hydrolyzed to **14**, a feature that was used in the NMR characterization of products. Potential anthraquinone biosynthesis is shown in Figure S26. The biosynthetic mechanisms leading to compounds **10** and **13** in echinoderms and fungi are shown in Figure S27.

The products of echinoderm PKSs SpPks1 and CrPKS do not necessarily represent the final pigment compounds found in the animals. In general, extensive oxidative tailoring and other modifications of the core polyketide scaffold are required, but since the genes are not clustered in echinoderms, further research is needed to identify these downstream steps.^28^ In terms of precursor polyketides, we previously showed that SpPks1 synthesizes ATHN (**10**), while here we show that CrPKS synthesizes precursors that may be processed into different pigment structural families within the animal (Figure 1). By incorporating butyrate, CrPKS directly synthesized the precursor to natural product **2**. The pigments with the nor-rubrofusarin scaffold such as **3** would originate from the dehydration of YWA1 (**13**) to produce YWA2 (**14**), a natural product that is isolated under some conditions (Figure 6). Tetrahydroxynaphthalene derivatives such as **1** might also be direct products of **11** and **12**, as hydrolytic side chain removal has been previously shown to afford this modification in fungi.^44^ The origin of anthraquinone pigments remains mysterious. We detected a trace of a compound with a mass consistent with anthraquinone in the pellet of CrPKS-expressing yeasts (Figure S26), suggesting the possibility that CrPKS or its relatives might make anthraquinones.

## Discussion

Here, we demonstrate that the crinoid PKS CrPKS produces YWA1 (**13**) and analogs, which are two carbons longer than those produced by the urchin PKS, SpPks1. Moreover, CrPKS had a different starter unit selectivity than SpPks1, using butyrate/ethylmalonate in addition to acetate/malonate. By comparing CrPKS with SpPks1, we identified specific residues in the KS lining the active-site pocket responsible for chain length and starter unit selection. Swapping the CrPKS-specific residues into SpPks1 led to the synthesis of the 14-carbon YWA1 (**13**) analogs, which were not made by the wild-type enzyme. Conversely, mutating the CrPKS residues led to an increase in the formation of 12-carbon polyketides.

This source of substrate and product selectivity is unusual in type I PKSs and reflects the unique qualities found in the animal PKS enzymes. For example, in the case of convergent evolution, fungal nonreducing PKSs (NRPKSs) synthesize similar or identical aromatic compounds as found in echinoderms.^11^ However, the NRPKSs have different evolutionary origins, domain architectures, and biochemical mechanisms in comparison to echinoderm PKSs (Figure S27). While mechanisms of chain length control have been studied in detail in NRPKSs, little is known about their function in the SpPks1 family. For example, while NRPKSs use the product template (PT) and thioesterase (TE) domains to control chain length and folding patterns, SpPks1 lacks these domains entirely.^7^^,^^8^^,^^9^^,^^10^^,^^11^^,^^12^^,^^13^^,^^45^ Overall, the mechanisms underlying these convergently evolved enzymes are quite different.

In summary, this study characterizes an enzyme underlying crinoid pigment synthesis and defines specific residues in the KS domain that are involved in starter unit and chain length determination. The results provide insight into the unique evolution of animal PKSs that will be useful in understanding the ecology and physiology of echinoderm pigments and provide a glimpse of the colorful palette of ancient marine life.

### Limitations of the study

Despite the discovery of important active site KS residues described here, we were still unable to use sequence differences to predict product structures within the echinoderms. This challenge possibly indicates the ancient vertical transmission of the echinoderm PKS enzymes, leading to a large number of mutations that reflect the phylogeny of the producing organisms, rather than enzyme function. It may be that characterizing many echinoderm PKSs from across the phylum could help in prediction. Since the echinoderm PKSs represent just a tiny fraction of the total animal PKS sequences available, this limitation also highlights the future scientific questions inherent in this widespread enzyme class.

## Resource availability

### Lead contact

Further information and requests for resources and reagents should be directed to and will be fulfilled by the lead contact, Eric Schmidt (ews1@utah.edu).

### Materials availability

Plasmids generated in this study are available upon request to Eric Schmidt (ews1@utah.edu).

### Data and code availability

• Data: All data are provided in the main article or supplemental information sections.
• Code: No unique code was written for this article.
• All other items: Raw data are available upon request to Eric Schmidt (ews1@utah.edu).

## Acknowledgments

This work was funded by 10.13039/100000001NSF CHE 2203613. We thank Dehai Li for providing the fungal strain and Bailey Miller for providing the photograph. Strain BJ5464-npgA and plasmid pxw55 were used with permission Yi Tang. NMR data were obtained at the University of Utah Health Sciences Center NMR Core Facility. We thank Chris Hill and Heidi Schubert for use of the microfluidizer.

## Author contributions

Conceptualization, E.W.S and F.L.; methodology, F.L. and Z.L.; investigation, F.L. and Z.L.; data analysis, F.L., Z.L., and E.W.S.; writing, E.W.S., F.L., and Z.L.; funding acquisition and project administration, E.W.S.

## Declaration of interests

The authors declare no competing interests.

## STAR★Methods

### Key resources table

**Table:** 

REAGENT or RESOURCE	SOURCE	IDENTIFIER
**Chemicals, peptides, and recombinant proteins**		

yeast synthetic drop-out media supplements without uracil	Sigma-Aldrich	Y1896-20G
yeast nitrogen base	Sigma-Aldrich	51483
yeast extract	Sigma-Aldrich	Y1625
peptone	Fisher Scientific	BP1420-2
glucose	Fisher Scientific	AC410950050
NaH_2_PO_4_	Fisher Scientific	BP329-500
imidazole	Fisher Scientific	AC122025000
Ni-NTA resin	GoldBio	H-350-100
NaCl	Fisher Scientific	S271-3
phosphate-buffered saline	Sigma-Aldrich	9005-64-5
acetyl-CoA	Coala Biosciences	SKU AC01
malonyl-CoA	Coala Biosciences	SKU MC01
NADPH	Medchemexpress	HY-F0003
SAM	Sigma-Aldrich	A7007-25MG
ethylmalonyl-CoA	Coala Biosciences	SKU EM01
butyryl-CoA	Coala Biosciences	SKU BC01
[13C3]-malonic acid	Cambridge Isotope	CLM-751-0.5
ATP	Sigma-Aldrich	A2754-500MG
MatB	This study	NA
2-[^13^C]-acetate	Sigma-Aldrich	282014-1G
22 Degrees Baume hydrochloric acid	Fisher Scientific	A142-212
TFA	Sigma-Aldrich	302031
Leucine enkephalin	Sigma-Aldrich	L9133-25MG

**Critical commercial assays**		

EasyComp™ Transformation Kit	Invitrogen	K505001
Amicon Ultra 100 MWCO centrifugal filters	EMD Millipore	UFC900308
Superose™ 6 Increase 10/300 GL column	Cytiva	29091596
Anti-DYKDDDDK Affinity Resin	Genscript	L00432
Acquity UPLC protein BEH C4 1.7 μm column (2.1 x 100 mm)	Waters	186004496
Luna 5 μm C18 (2) LC column (250 Å∼ 10 mm)	Phenomenex	NA

**Experimental models: Organisms/strains**		

*Saccharomyces cerevisiae*	Lee, K. K. M. et al.^46^	BJ5464-npgA
*Aspergillus nidulans*	Zhang, K. et al.^37^	A1145+PKS47G

**Oligonucleotides**		

Primers for PKS gene mutant, see Table S1	This paper	NA

**Recombinant DNA**		

Plasmid pxw55-CrPKS	This paper	NA
Plasmid pxw55-SpPks1	Li, F. et al.^4^	NA
Plasmid pxw55-SpPks1-GLMD	This paper	NA
Plasmid pxw55-CrPKS-GMMD	This paper	NA

**Software and algorithms**		

Prism 8	GraphPad Prism version 8.4.3 for Mac OS^47^	www.graphpad.com
AlphaFold	Jumper, J. et al.^43^	NA
PyMOL	Schrodinger LLC.^48^	NA

### Experimental model and study participant details

The following microbial strains were used, obtained from Nancy DaSilva and Dehai Li, respectively:

*Saccharomyces cerevisiae* BJ5464-npgA.

Aspergillus nidulans A1145+PKS47G.

### Method details

#### Plasmid construction and analysis

Plasmid pxw55-CrPKS was synthesized by GENEWIZ with N-FLAG and C-His tags at SpeI and PmlI. pxw55-CrPKS-GMMD was generated from wild-type pxw55-CrPKS using primers Cr-GMMD-f and Cr-GMMD-r, while pxw55-SpPks1 used primers Sp-GLMD-f and Sp-GLMD-r. Plasmids were verified by Plasmidsaurus sequencing.

#### Protein expression

Plasmids were transformed into *Saccharomyces cerevisiae* BJ5464-*npgA* (MATα ura3-52 trp1 leu2-Δ1 his3Δ200 pep::HIS3 prb1d1.6R can1 GAL) using S.c. EasyComp™ Transformation Kit (Invitrogen, K505001). Colonies appeared on selected uracil-deficient agar (1.39 g/L yeast synthetic drop-out media supplements without uracil (Sigma-Aldrich), 6.7 g/L yeast nitrogen base (Sigma-Aldrich), 40 mL/L 50% glucose solution, 20 g/L agar) and incubated at 30°C for 48 h.

#### Protein purification

Three single colonies of *S. cerevisiae* BJ5464-*npgA* containing recombinant plasmid were used to inoculate seed cultures in uracil-deficient broth (5 mL; 1.39 g/L yeast synthetic drop-out media supplements without uracil (Sigma-Aldrich), 6.7 g/L yeast nitrogen base (Sigma-Aldrich), 40 mL/L 50% glucose solution)) at 30°C with shaking at 180 rpm for 24 h. The seed culture (1 mL/L) was added to yeast peptone dextrose broth (10 g/L yeast extract, 20 g/L peptone, 20 g/L glucose). The 6 × 1 L culture was grown at 28°C with shaking at 180 rpm. After 72 h, the cells were pelleted at 3,214 × *g* for 20 min at 4°C.

Cell pellets were resuspended in lysis buffer (50 mM NaH_2_PO_4_, 150 mM NaCl, 10 mM imidazole, pH 8.0). The suspension was lysed by passing three times through a microfluidizer (LM20 Microfluidizer™ Processor) set at 20,000 psi. The resulting mixture was centrifuged at 28,928 × *g* for 40 min at 4°C to separate supernatant from cell debris. The supernatant was incubated with Ni-NTA resin for 3h at 4°C on a rotary shaker. After incubation, the Ni-NTA resin was pooled into an open column. The resin was washed with 25 mL each of 20 mM and 50 mM imidazole in 50 mM Tris-HCl buffer (500 mM NaCl, pH 8.0). CrPKS was eluted by 3 × 5 mL 250 mM imidazole in sodium phosphate buffer (50 mM NaH_2_PO_4_, 150 mM NaCl, pH 8.0). Eluted CrPKS fraction was concentrated to 1 mL, buffer exchanged (15 mL of 100 mM NaH_2_PO_4_, 100 mM NaCl, pH 8.0) and further concentrated to 1 mL final volume using Amicon Ultra 100 MWCO centrifugal filters (EMD Millipore).

Crude enzyme was injected onto a Superose™ 6 Increase 10/300 GL column and eluted with sodium phosphate buffer (100 mM sodium phosphate, 100 mM NaCl, pH 7.5). The dimeric enzyme eluted at 12-14 mL. Following sizing, anti-DYKDDDDK Affinity Resin (Pierce A36801) was used to further purify CrPKS/CrPKS-GMMD. The usage of Antiflag resin followed the manufacturer’s instruction. CrPKS/CrPKS-GMMD was eluted twice with 500 μL elution buffer (3 × DYKDDDDK peptide,1.5 mg/mL) in phosphate-buffered saline (Sigma-Aldrich 9005-64-5) to remove impurities from CrPKS/CrPKS-GMMD. Purified CrPKS/CrPKS-GMMD was concentrated to 100 μL using Amicon Ultra 100 MWCO centrifugal filters (EMD Millipore), then it was repeatedly diluted to 500 μL by buffer (100 mM sodium phosphate buffer, 100 mM NaCl, pH 7.5) and concentrated to 100 μL. CrPKS was obtained (estimated using UV-280 spectroscopy (1 ABS=1mg/mL method) on a NanoDrop instrument) at 3 mg/L, CrPKS-GMMD at 3.2 mg/mL.

#### Initial characterization of CrPKS

Purified CrPKS (5 μM) was incubated with the mixture of 2 mM acetyl-CoA and 2 mM malonyl-CoA, or only 2 mM malonyl-CoA and with or without 1 mM NADPH and 1 mM SAM in 100 mM sodium phosphate pH 7.5 for 12 h at 22°C. Reactions were quenched with the same volume of acetonitrile and analyzed as described in the analysis section. No different products were detected between the reactions with or without NADPH as well the reactions of with or without acetyl-CoA. This was confirmed in triplicate reactions.

#### Acyl substrate specificity assays

Ethylmalonyl-CoA (2 mM) or butyryl-CoA (2 mM) were incubated with CrPKS (5 μM) in sodium phosphate (100 mM sodium phosphate, 100 mM NaCl, pH =7.5) with addition of malonyl-CoA (2 mM). Three replicates were used for each CoA substrate. Reaction mixtures were incubated at 22°C for 12 h before analysis by LCMS.

#### ^13^C_3_ malonyl-CoA incorporation assay

Reaction mixtures (20 μL) containing 5 mM [^13^C_3_]-malonic acid, 5 mM MgCl_2_, 5 mM ATP, 5 mM CoA, 10 μM MatB^49^ and 5 μM CrPKS in sodium phosphate (100 mM sodium phosphate, 100 mM NaCl, pH =7.5) with 2 mM of other starter units (acetyl-CoA, ethylmalonyl-CoA or butyryl-CoA) were incubated at 22°C for 12 h before analysis by LCMS. (The enzyme MatB was included because it uses ATP and malonate to synthesize malonyl-CoA *in situ*). When butyryl-CoA and ethylmalonyl-CoA were added, we observed *m/z* 289.1022 for YWA1 and 271.1094 for compound **18**. The experiments were performed in triplicate.

Yeast pellet experiments for SpPks1, CrPKS, SpPks1-GLMD, CrPKS-GMMD. *S. cerevisiae* expressing SpPks1, CrPKS, CrPKS-GMMD and SpPks1-GLMD were grown for 3 days in 1L YPD medium (Difco) at 28°C, 180 rpm. A portion (50 mL) of each culture was centrifuged at 4,500 × *g* to collect the cell pellet. The pellet was washed twice with sodium phosphate buffer (100 mM sodium phosphate, 100 mM NaCl, pH =7.5) and resuspended in 2 mL sodium phosphate buffer. A final concentration of 5 mM acetate solution (^13^C_1_-acetate:acetate = 1:1) was added to resuspended pellet. Cells were then left to incubate for 18 h at room temperature on a rotary shaker. Cells were obtained by centrifugation at 28,928 × *g* for 1 min. The resulting pellets were washed with acetonitrile (1 mL) and centrifuged at 28,928 × *g* for 10 min to yield a soluble fraction, of which 2 μL was used for LC-MS analysis. Each reaction was performed in triplicate.

#### Michaelis-Menten kinetics for CrPKS wild-type and mutant

Based upon the time course experiment, a reaction time of 120 min was chosen for kinetics analysis, using the following condition: 8 μM PKS in sodium phosphate buffer, pH 7.5 22°C for 2 h, variable substrate concentrations. Reactions were quenched with acetonitrile (20 μL) and analyzed by mass spectrometry as described above. Each condition was performed using triplicate replicates, and data were analyzed using Prism 8^47^ with a nonlinear regression-Michaelis-Menten method.

#### Mass spectrometric analysis

Enzymatic reactions were quenched and precipitated by equal volume acetonitrile and centrifuged for 10 min at 28,928 × *g*, then used for mass spectrometry. The resulting supernatants (5 μL was injected following enzyme reactions; 2 μL following pellet experiments) were run on an Acquity UPLC protein BEH C4 1.7 μm column (2.1 x 100 mm) with a linear gradient of 5-100% mobile phase B over 10 min (mobile phase A: H_2_O with 0.01 % formic acid; mobile phase B: MeCN), flowrate of 0.4 mL/min. Mass spectrometry of enzyme and synthetic reaction products as well as pellet experiments were done in negative mode with the following parameters: detected range: 100-1000 Da, scan time: 0.5 s, data format: centroid. YWA1 was analyzed by different voltage with following parameters: detected range: 100-1000 Da, scan time: 0.5 s, data format: centroid, collision voltage: 30V.

#### pH optimization of CrPKS reactions

All enzyme reactions were performed at 22°C unless otherwise stated. CrPKS (2 μL, 7 mg/mL) was added to 10 μL sodium phosphate (100 mM sodium phosphate, 100 mM NaCl) with pH at 7.0, 7.5, and 8.0. Sodium phosphate (100 mM sodium phosphate, 100 mM NaCl) pH = 8.0 was selected. Conditions were further optimized with butyryl-CoA, using CrPKS (2 μL, 7mg/mL) incubated with malonyl-CoA (2 mM) and butyryl-CoA (2 mM) in sodium phosphate (100 mM sodium phosphate, 100 mM NaCl). Following the analysis, pH 7.5 was used in all further experiments. Optimization experiments were done in triplicate.

#### Large-scale reaction with CrPKS

CrPKS was expressed in 18 L culture, and cells were pelleted and used to purify CrPKS. The fraction that eluted in 3×5 mL 250 mM imidazole in sodium phosphate buffer (50 mM NaH_2_PO_4_, 150 mM NaCl, pH 8.0) was dialyzed into sodium phosphate (100 mM sodium phosphate, 100 mM NaCl, pH =7.5). The enzyme was incubated with 5 mM malonic acid, 5 mM MgCl_2_, 5 mM ATP, 5 mM CoA, 10 μM MatB.^49^^,^^50^ After 3 and 6 h, an additional 20 mg and 60 mg ATP were added, respectively. The mixture was stirred for an additional 10 h, for a total incubation time of 16 h. The reaction mixture was extracted with 3 × 50 mL ethyl acetate. The ethyl acetate was dried by sodium sulfate and concentrated under reduced pressure. The product was purified using HPLC (Thermo Scientific, UltiMate 3000) with Luna 5 μm C18 (2) LC column (250 × 10 mm) with a linear gradient of 30-100% mobile phase B over 20 min (solvent A, H_2_O with 0.1% TFA; solvent B, MeCN) at a flow of 3.5 mL/min; t_R_=12.5 min.

#### ^13^C_1_ acetyl-CoA incorporation assay

Reaction mixtures (20 μL) containing 2 mM malonyl-CoA, 5 mM 2-[^13^C_1_]-acetate, 5 mM MgCl_2_, 5 mM ATP, 5 mM CoA, 10 μM enzyme ACS^50^ and 5 μM CrPKS in sodium phosphate (100 mM sodium phosphate, 100 mM NaCl, pH =7.5) were incubated at 22°C for 12 h before analysis by LCMS. (The enzyme ACS was included because it uses ATP and acetate to synthesize acetyl-CoA *in situ*). New ions were observed at 1 Da (*m/z* 276.0623) greater than their unlabeled counterparts showing incorporation of one acetate.

#### Comparison of enzyme reaction products with YWA1 (13)

The fungus *Aspergillus nidulans* A1145+PKS47G was grown and extracted as previously described to provide **13** and its derivatives as a mixture.^37^ These were compared with the ^13^C-malonate-labeled CrPKS enzyme reaction products generated as described above.

To confirm that **13** and relatives were present in the fungal extract, a dried aliquot (500 mg) containing **13** was stirred in 22 Degrees Baume hydrochloric acid (5 mL) overnight under a blanket of argon gas. A CombiFlash (NEXTgen 300+) was used with the procedure (HP C18 column, flowrate 30 mL/min, solvent acetonitrile and water, gradient 5% acetonitrile to 100% in 10 min, **14** eluted at 5 min) to yield purified **14** (50 mg). ^1^H and ^13^C NMR confirmed the identity and purity of **14** (Figures S8 and S9).

#### Structure determination of butyrated compounds 17 and 18

High-resolution MS of reaction products confirmed the molecular formulae of products with butyrate starter units. Enzymatic incorporation of ^13^C_3_-malonyl-CoA with unlabeled butyryl-CoA was used to further confirm the incorporation of butyrate. The resulting products **17** and **18** are 10 Da heavier than product resulting from both unlabeled substrates, indicating that 5 units of malonyl-CoA and 1 unit of butyrl-CoA are incorporated (Figures S14 and S15). This left two possible structures, based upon known enzymology: a pyrone, and a naphthalene. To differentiate between these possibilities, MS fragmentation and comparison of labeled versus unlabeled products (Figures S14 and S15) revealed that the fragment ions are also heavier by 10 Da in the labeled compounds in comparison to the unlabeled compounds, supporting the presence of butyrate as a side chain. The fragmentation results are very similar to those shown for compound **16** (Figure S10), which contains the all-malonate sidechain, and which was experimentally validated in comparison to authentic standards. Thus, the available data supported the structures **17** and **18** as drawn.

#### CrPKS wild-type and mutant time course experiment

PKS proteins (8 μM) were incubated with substrate in sodium phosphate buffer, pH 7.5 at 22°C for 2 h. For time course experiments, malonyl-CoA (2 mM) and butyryl-CoA (2 mM) were added to reaction mixtures. Reactions were quenched at 30, 60, 120, and 180 min and analyzed by UPLC-MS. Area under the curve (AUC) determined with an internal standard (1 ng/μL Leucine enkephalin) was used to quantify products. Each time point was performed triplicate.

### Quantification and statistical analysis

1. All LC-MS raw data was analyzed by MassLynx. Prism 8^47^ was used to generate figures including: all chromatography figures in the main text; and SI figures that are not in the raw form.
2. Structures of SpPks1-KS, SpPks1-GLMD-KS, CrPKS-KS and CrPKS-GMMD-KS were modelled using AlphaFold^43^ and analyzed with PyMOL.^51^
