# Supplementary material for: Molecular basis of pigment structural diversity in echinoderms
Source: iScience. 2024 Aug 30;27(9):110834. doi: 10.1016/j.isci.2024.110834 (PMC11414698; doi:10.1016/j.isci.2024.110834)
Supplement: Document S1. Figures S1–S27 and Table S1 [file mmc1.pdf]

iScience, Volume *27*

Supplemental information

Molecular basis of pigment structural diversity in echinoderms

Feng Li, Zhenjian Lin, and Eric W. Schmidt

Supporting Table and Figures.

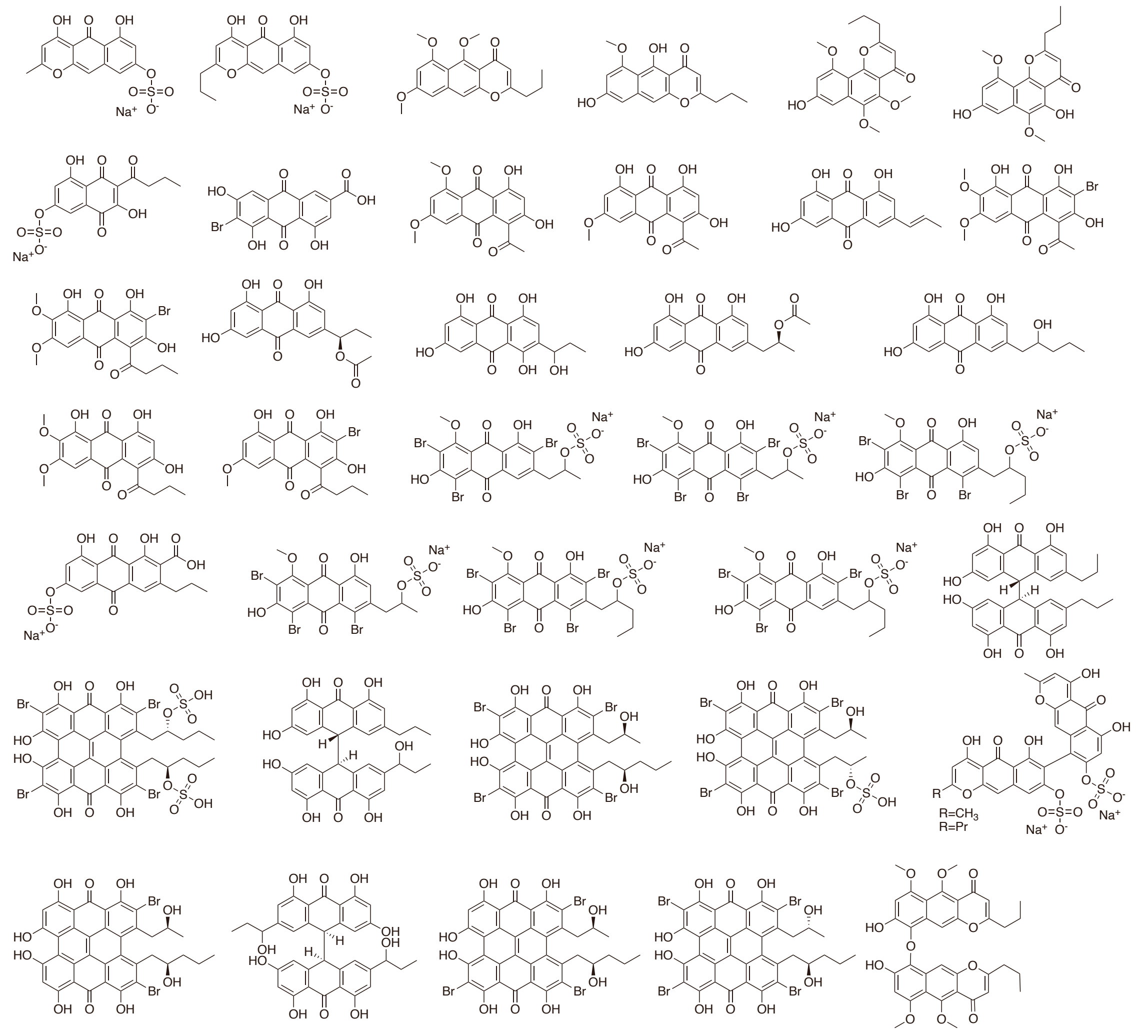

Figure S1: Pigments previously reported from Crinoidea, related to Figure 1 and the Introduction.

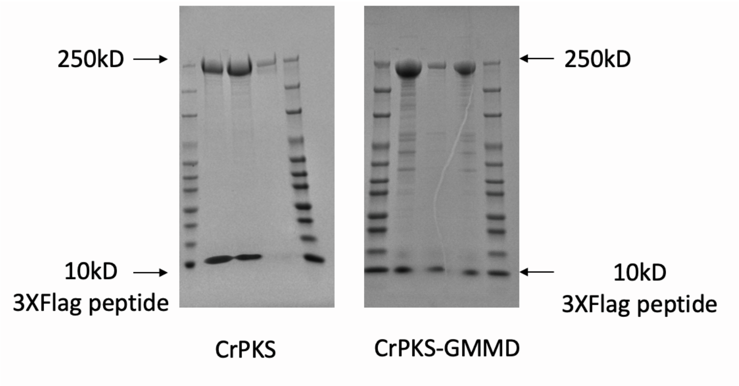

Figure S2: Purification of CrPKS and CrPKS-GMMD From BJ5464-NpgA, related to Figure 3 and STAR Methods. SDS-PAGE of soluble CrPKS and CrPKS-GMMD recovered using Ni-NTA resin followed purification by FPLC and anti-FLAG resin.

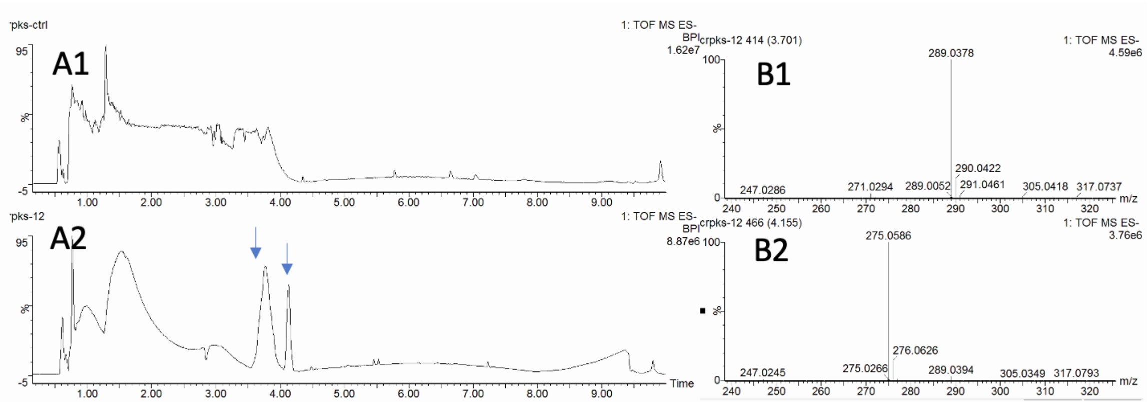

Figure S3: LC-MS chromatograms of CrPKS enzyme assay with malonyl-CoA, related to Figure 3. A) Base-peak intensity ion chromatogram of assay (A2), compared to boiled enzyme control (A1); B) Peaks shown with blue arrows had an observed *m/z* at 275.0586 and 289.0378, corresponding to known compounds **13** and **16**, respectively.

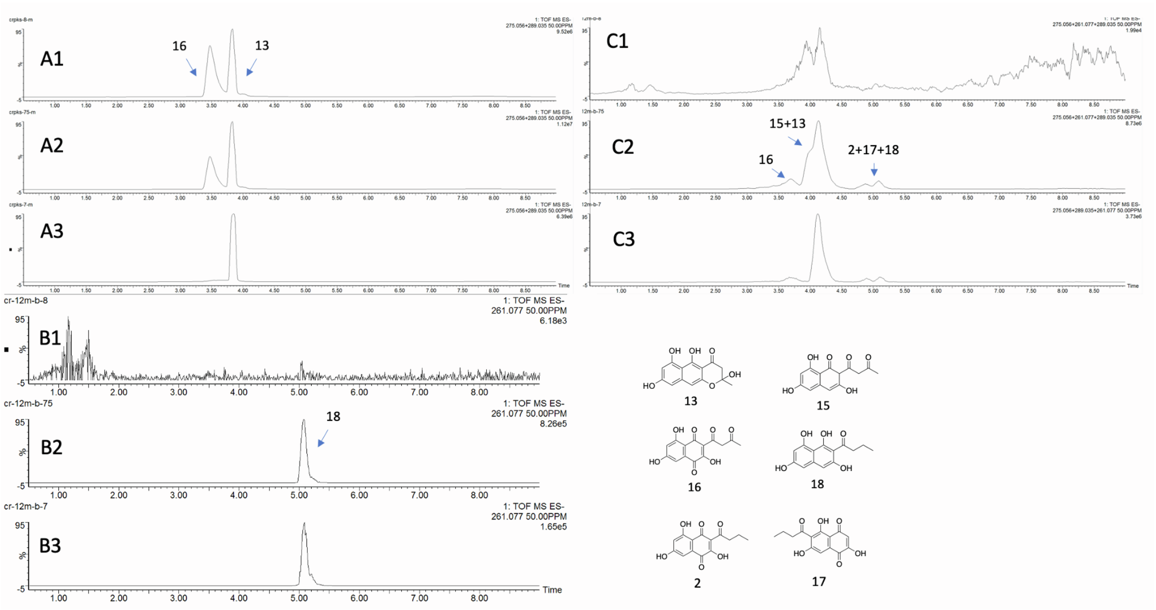

Figure S4: Optimization of CrPKS reactions, related to Figure 3 and STAR Methods. A) Extracted ion chromatograms of **13** and **16** for CrPKS incubated with malonyl-CoA in sodium phosphate buffer at (A1) pH = 8, (A2) pH = 7.5, (A3) pH = 7.0. B) Extracted ion chromatograms of **18** for CrPKS incubated with malonyl-CoA and butyryl-CoA in sodium phosphate buffer at (B1) pH = 8, (B2) pH = 7.5, (B3) pH = 7.0. C) Extracted ion chromatograms of **18**+**13**+**16**+**2**+**17** for CrPKS incubated with malonyl-CoA and butyryl-CoA in sodium phosphate buffer at (C1) pH = 8, (C2) pH = 7.5, (C3) pH = 7.0.

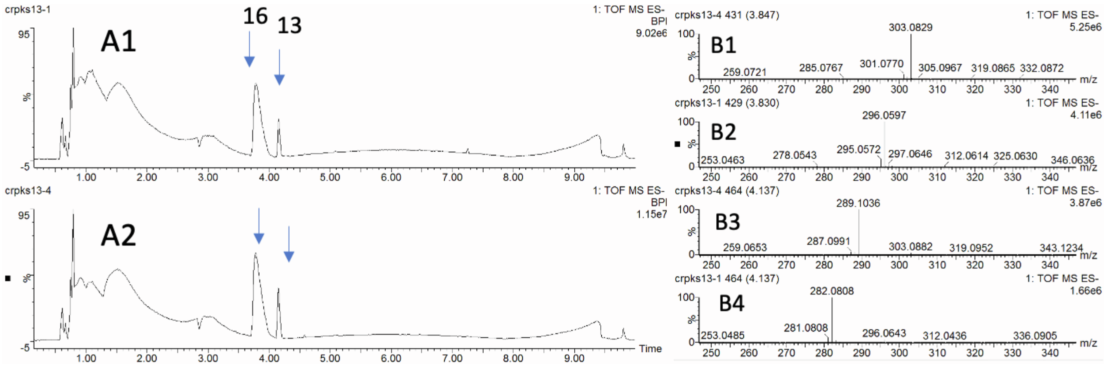

Figure S5: CrPKS incorporates 7 malonate units, related to Figure 3 and STAR Methods. A) CrPKS incubated with (A1) $^{13}C_1$-malonyl-CoA and (A2) $^{13}C_3$-malonyl-CoA. B) Molecular ions for **16** from (B1) $^{13}C_3$-malonyl-CoA and (B2) $^{13}C_1$-malonyl-CoA; molecular ions for **13** from (B3) $^{13}C_3$-malonyl-CoA and (B4)$^{13}C_1$-malonyl-CoA.

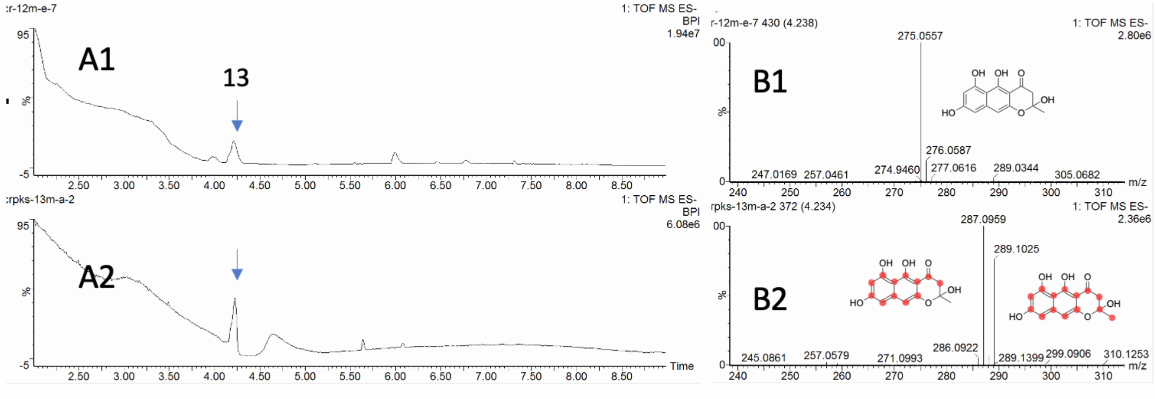

Figure S6: CrPKS uses both acetyl- and malonyl-CoA as starter units, related to Figure 3 and STAR Methods. A) Base peak intensity chromatograms of CrPKS incubated with (A1) malonyl-CoA, (A2) acetyl-CoA and $^{13}C_3$-malonyl-CoA. B) Molecular ions of compound **13** from (A1) and (A2).

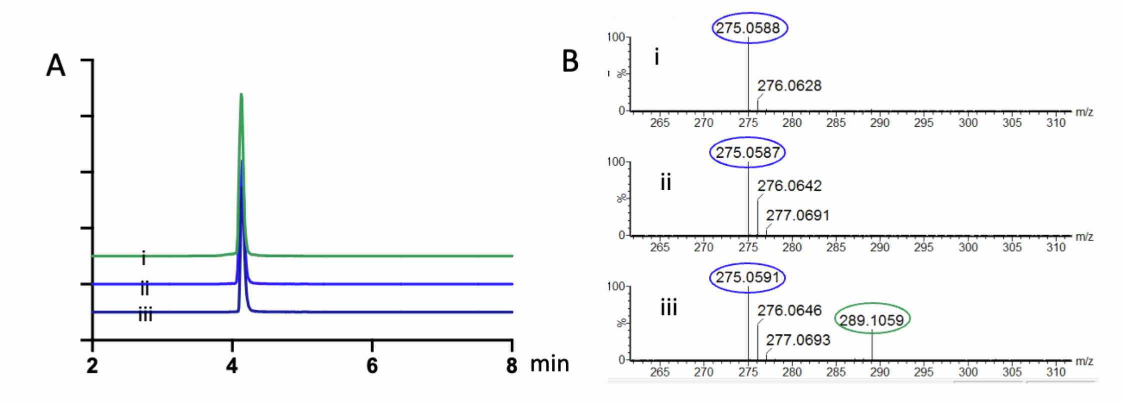

Figure S7: LC-MS chromatograms of CrPKS enzyme assay aligned with YWA1 from fungus, related to Figure 3. A) Extracted ion (*m/z* 275.0561) chromatograms and B) mass spectra. (i) Product of CrPKS reaction with malonyl-CoA, (ii) YWA1 from fungus, (iii) co-injection of YWA1 from fungus and product of CrPKS reaction when incubated with $^{13}C_3$-malonyl-CoA.

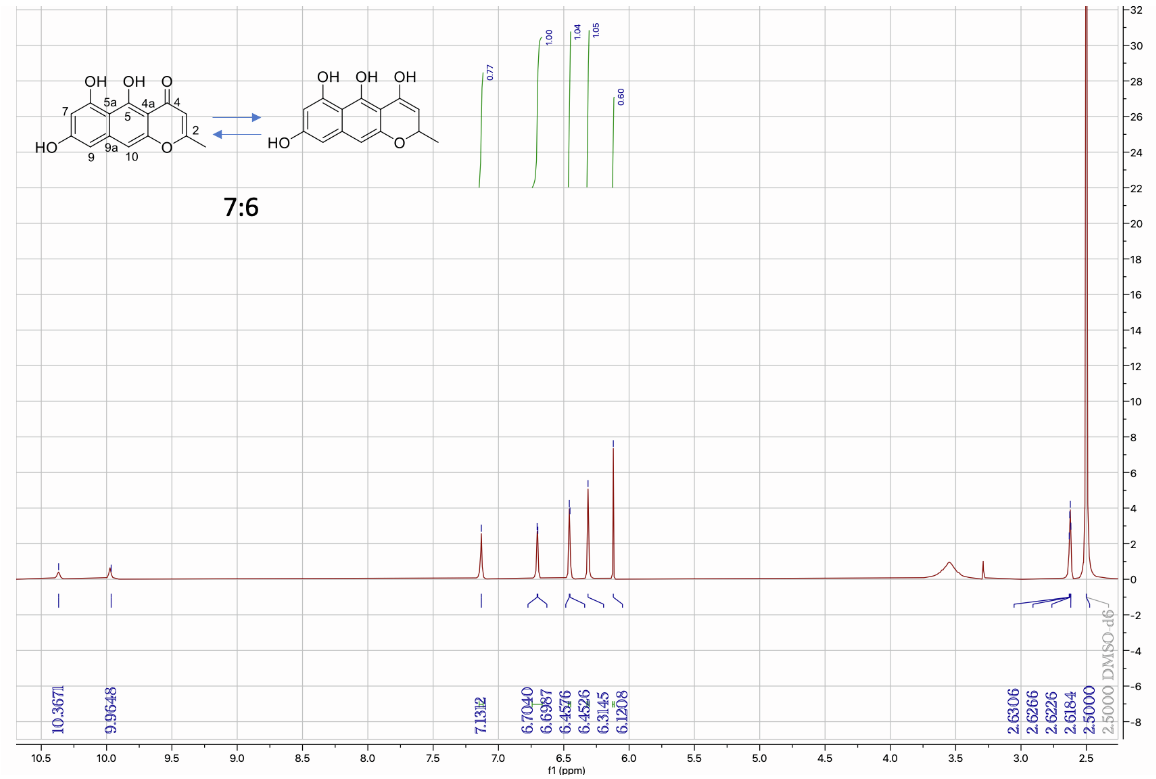

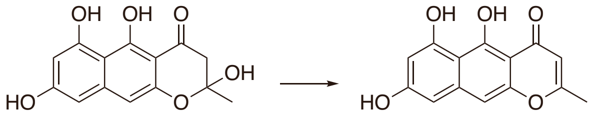

Figure S8: ^{1}H NMR of YWA2 (14), related to Figure 3 and STAR Methods. Top: NMR spectrum. Bottom: Conversion of 13 to 14 was accomplished synthetically in acidic conditions.

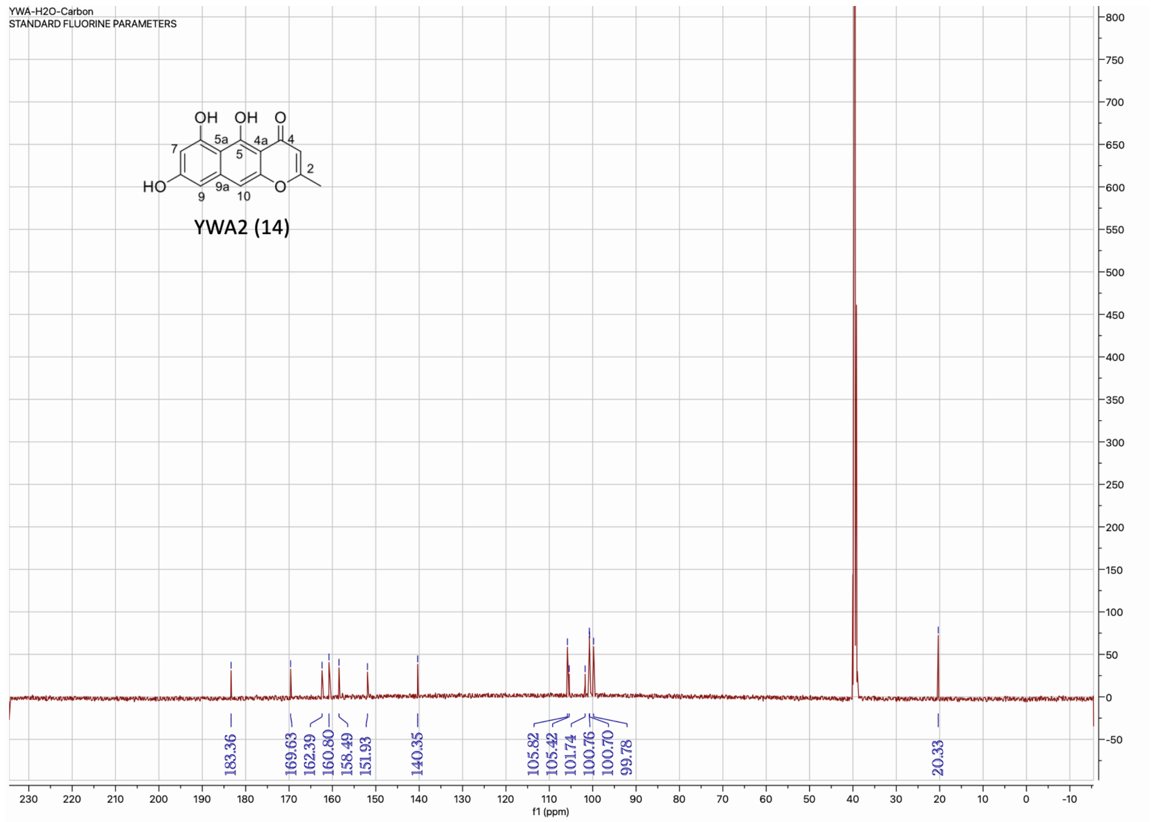

Figure S9: ^{13}C NMR of YWA2 (14), related to Figure 3 and STAR Methods.

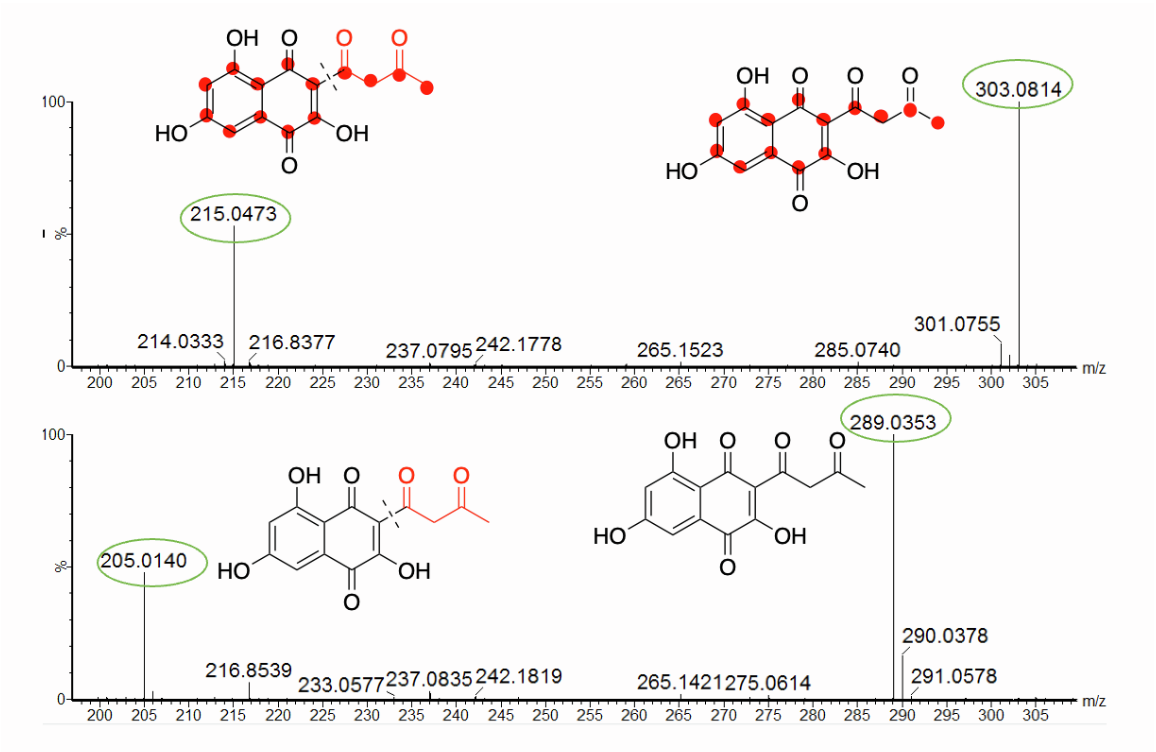

Figure S10: MS fragments of compound 16, related to Figure 3. (Upper) $^{13}C_3$-malonyl-CoA incorporated products. (Lower) Unlabeled products.

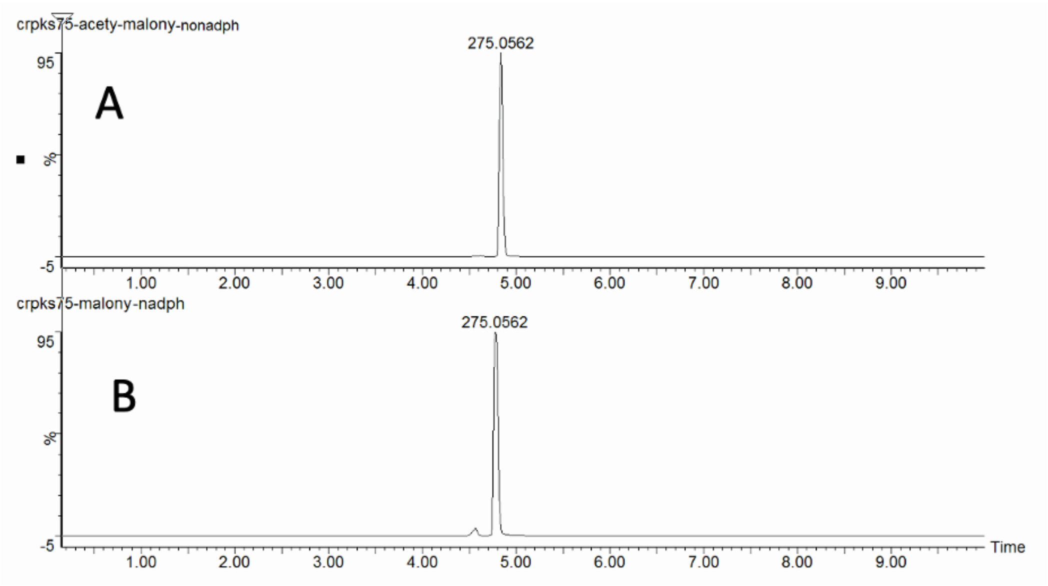

Figure S11: NADPH does not alter CrPKS product profile, related to Figure 3 and STAR Methods. CrPKS incubated with 2 mM malonyl-CoA A) without NADPH; B) with NADPH.

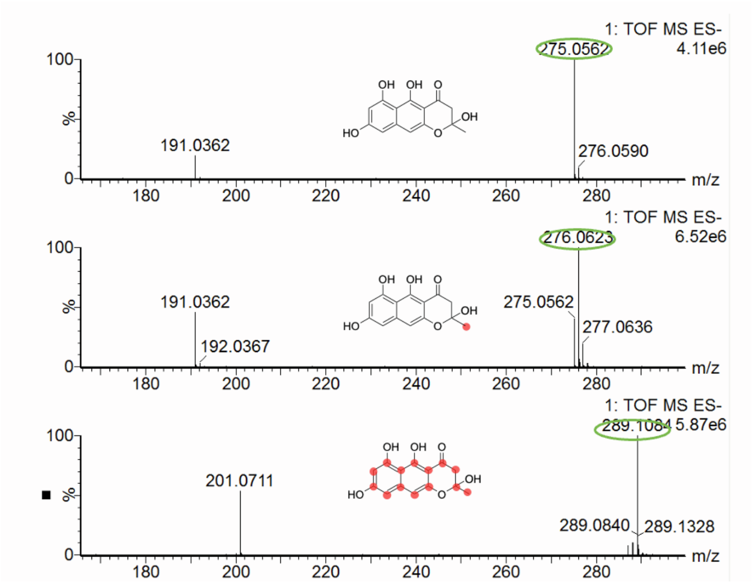

Figure S12: $^{13}C_1$-acetyl-CoA incorporation experiments, related to Figure 3 and STAR Methods. CrPKS incubated with A) malonyl-CoA, B) $^{13}C_1$-acetyl-CoA, C) $^{13}C_3$ malonyl-CoA.

.

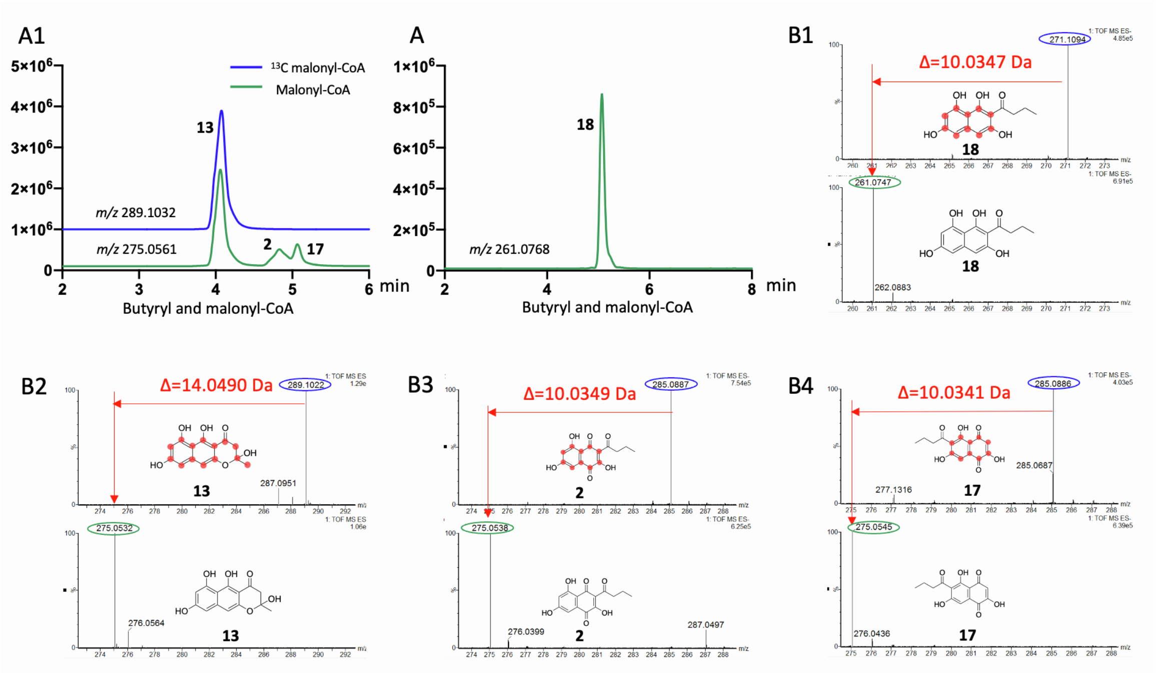

Figure S13: LC-MS chromatograms of CrPKS enzyme assay with malonyl-CoA and butyryl-CoA, related to Figure 4 and STAR Methods. A) New peaks were detected when CrPKS was incubated with a mixture of malonyl-CoA and butyryl-CoA, seen at *m/z* 275.0651. B) Molecular ions of the new peak, showing results when CrPKS was incubated with butyryl-CoA and either malonyl- or $^{13}C_3$-malonyl-CoA. Five units of malonate were incorporated in the new products.

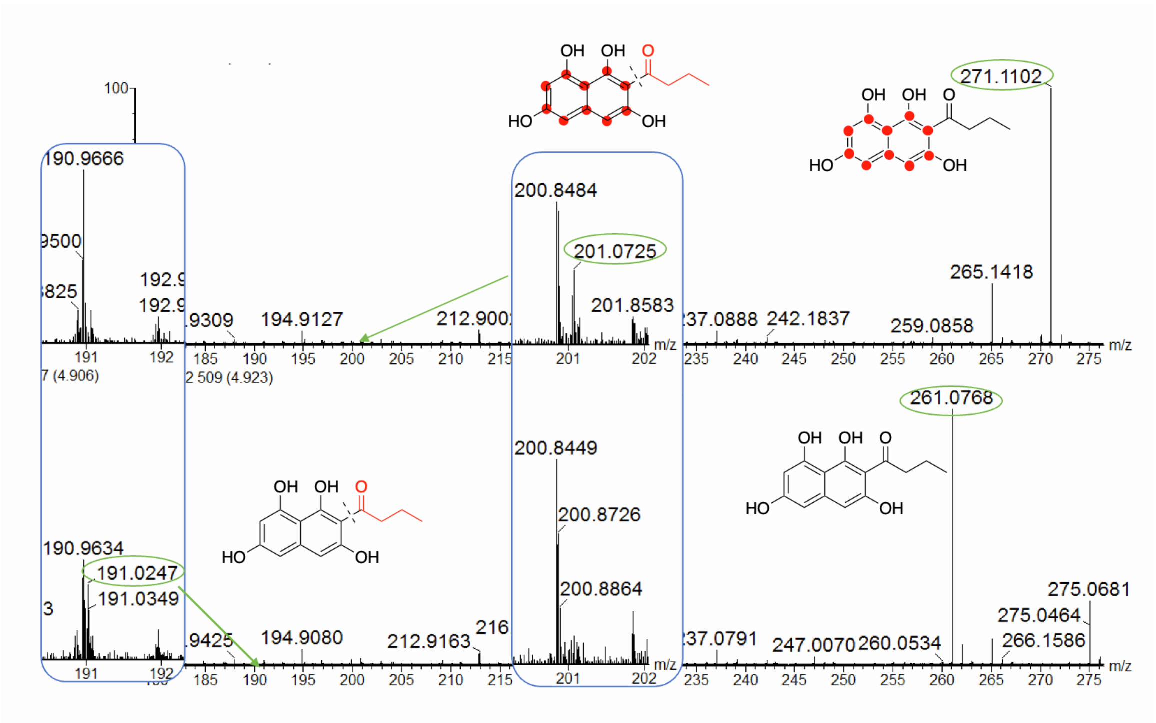

Figure S14: Fragments of compound 18, related to Figure 4. (Upper) $^{13}C_3$-malonyl-CoA incorporated products. (Lower) Unlabeled compounds.

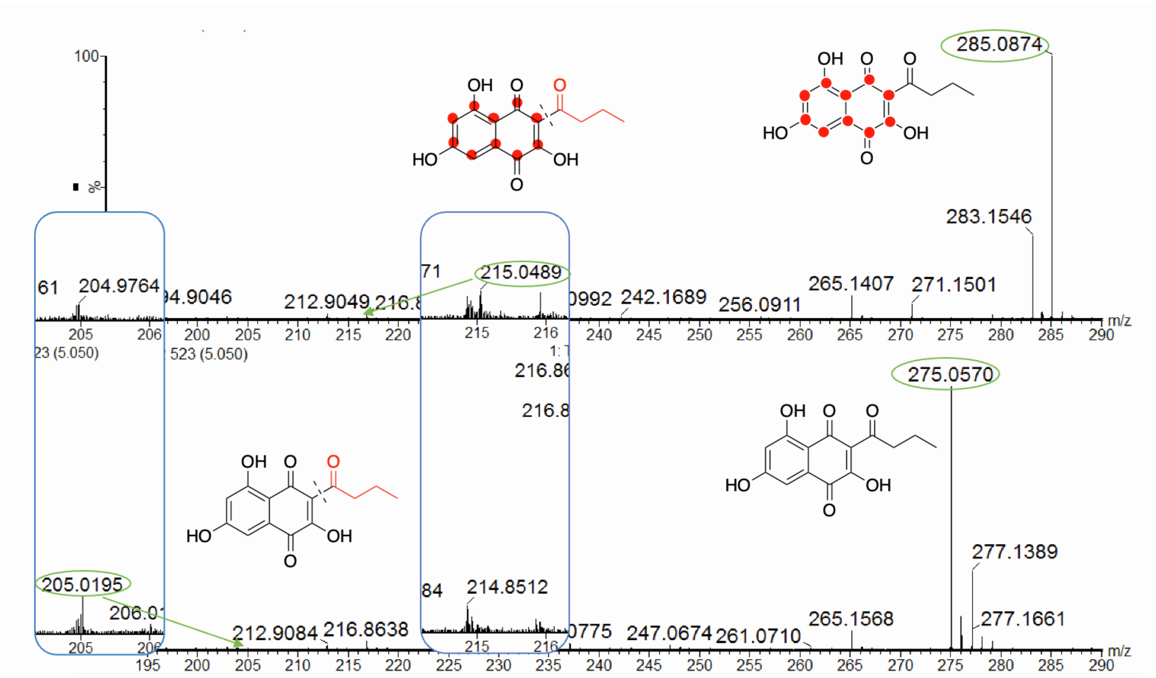

Figure S15: Fragments of compound 2 (or 17), related to Figure 4. (Upper) $^{13}C_3$-malonyl-CoA incorporated products. (Lower) Unlabeled compounds.

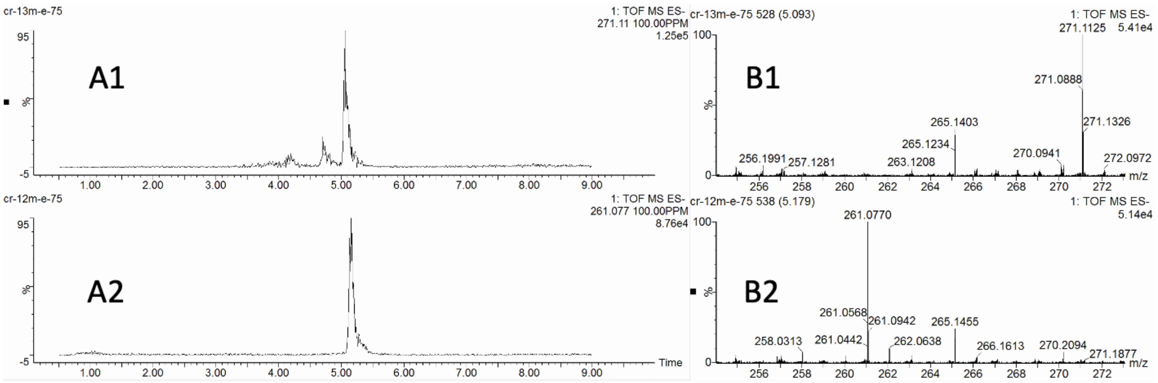

Figure S16: CrPKS uses ethymalonyl-CoA as starter unit, related to Figure 4 and STAR Methods. A) Extracted ion chromatogram of (A1) *m/z* = 271.1104 in reaction incubated with $^{13}C_3$-malonyl-CoA and ethymalonyl-CoA, (A2) *m/z* = 261.0868 in reaction incubated with malonyl-CoA and ethymalonyl-CoA. B) Mass spectra of ethylmalonyl-CoA assay product showing one ethylmalonyl-CoA and 5 malonyl-CoA used.

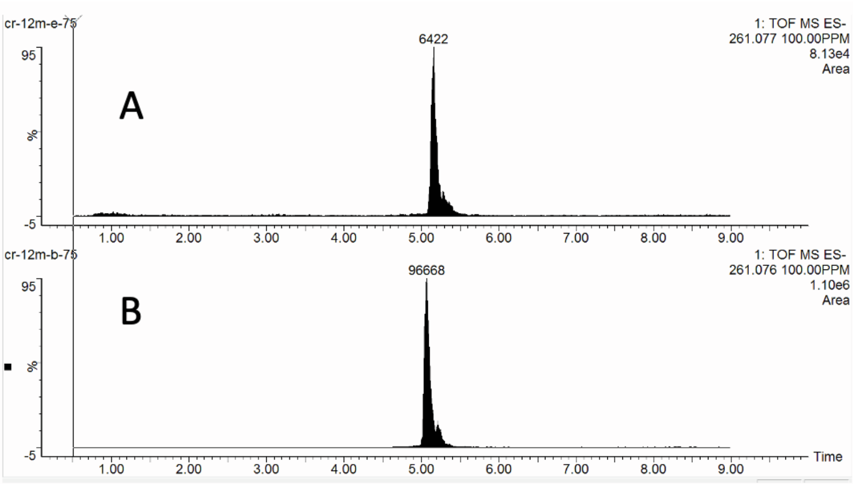

Figure S17: Butyryl-CoA is a preferable starter unit in comparison to ethylmalonyl-CoA, related to Figure 4 and STAR Methods. CrPKS incubated with malonyl-CoA and A) ethylmalony-CoA; B) butyryl-CoA. The number above the peak indicates the area under curve.

.

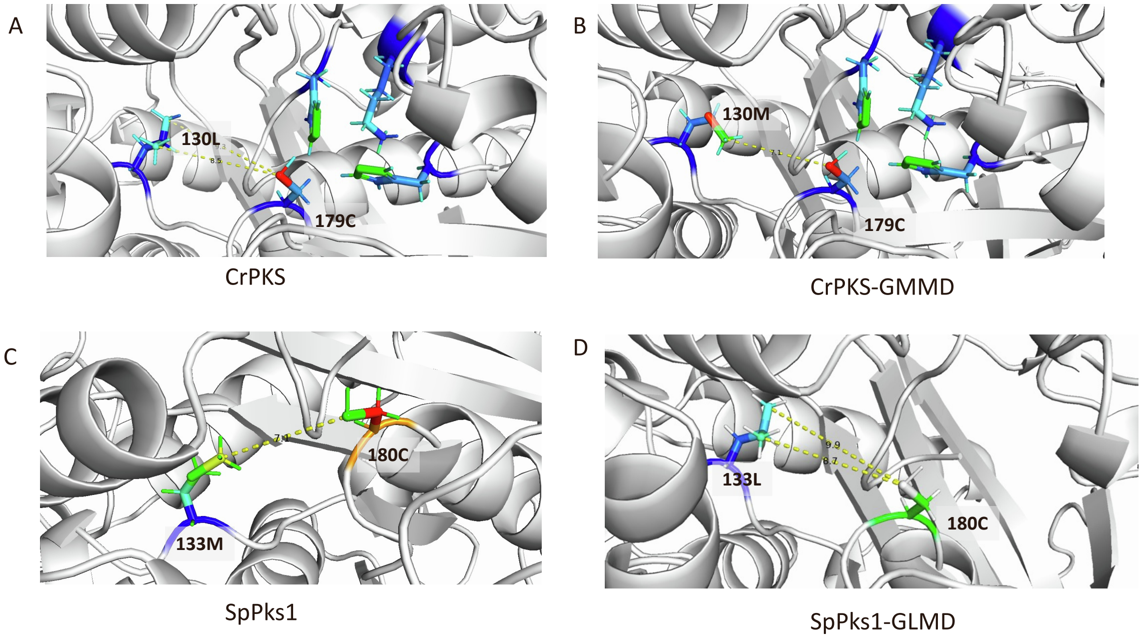

Figure S18: Mutation changes pocket size of PKS, related to Figures 5I and 5J. Predicted protein structure for A) CrPKS and B) CrPKS-GMMD, C) SpPks1 and D) SpPks1-GLMD.

.

.

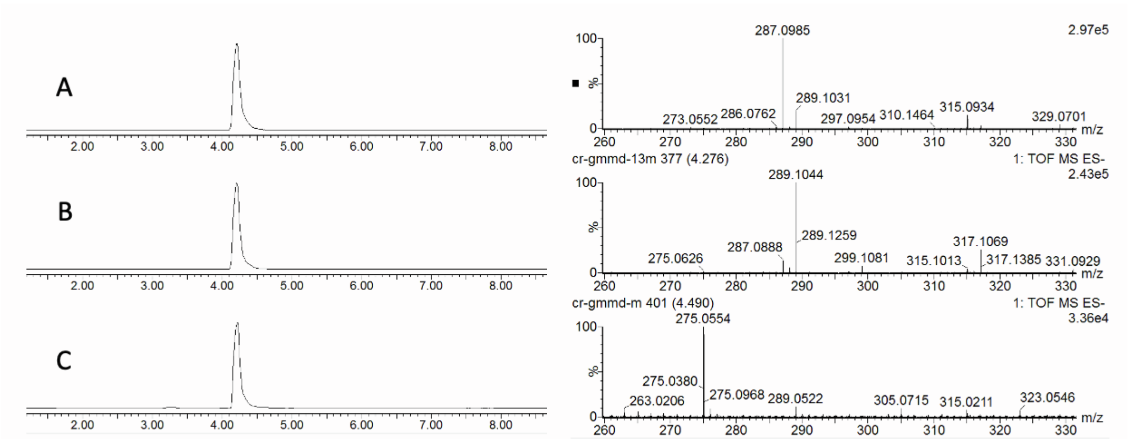

Figure S19: CrPKS-GMMD uses both acetyl-CoA and malonyl-CoA as starter units, related to Figure 5 and STAR Methods. Extracted ion chromatograms for YWA1 (**13**) from CrPKS-GMMD incubated with A) acetyl-CoA and $^{13}C_3$-malonyl-CoA, B) $^{13}C_3$-malonyl-CoA, C) malonyl-CoA.

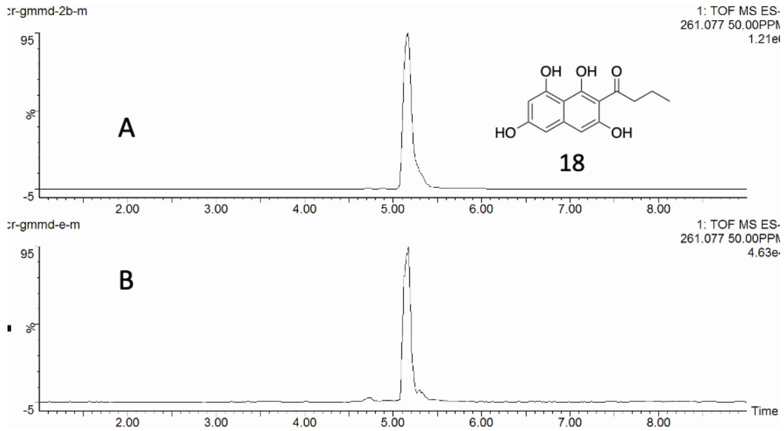

Figure S20: CrPKS-GMMD uses both butyryl-CoA and ethymalonyl-CoA starter units to synthesize compound 18, related to Figure 5 and STAR Methods. Extracted ion chromatograms of compound **18** from CrPKS incubated with malonyl-CoA and A) butyryl-CoA, B) ethylmalonyl-CoA. A smaller amount of compound **18** was synthesized with ethylmalonyl-CoA than with butyryl-CoA.

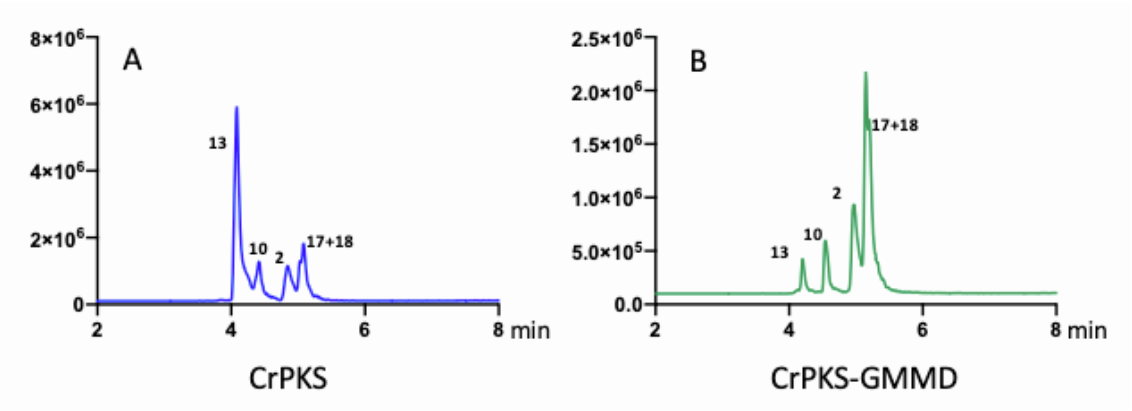

Figure S21: Extracted ion chromatograms of 2, 10, 12, 13 and 18, related to Figure 5. A) CrPKS, B) CrPKS-GMMD incubated with malonyl-CoA and butyryl-CoA. Chromatograms were extracted with *m/z* 275.0561(**13**+**2**/**17**), 233.0455 (**10**), 247.0421 (**12** overlapped with other peaks), 261.0768 (**18**) using error 20 ppm.

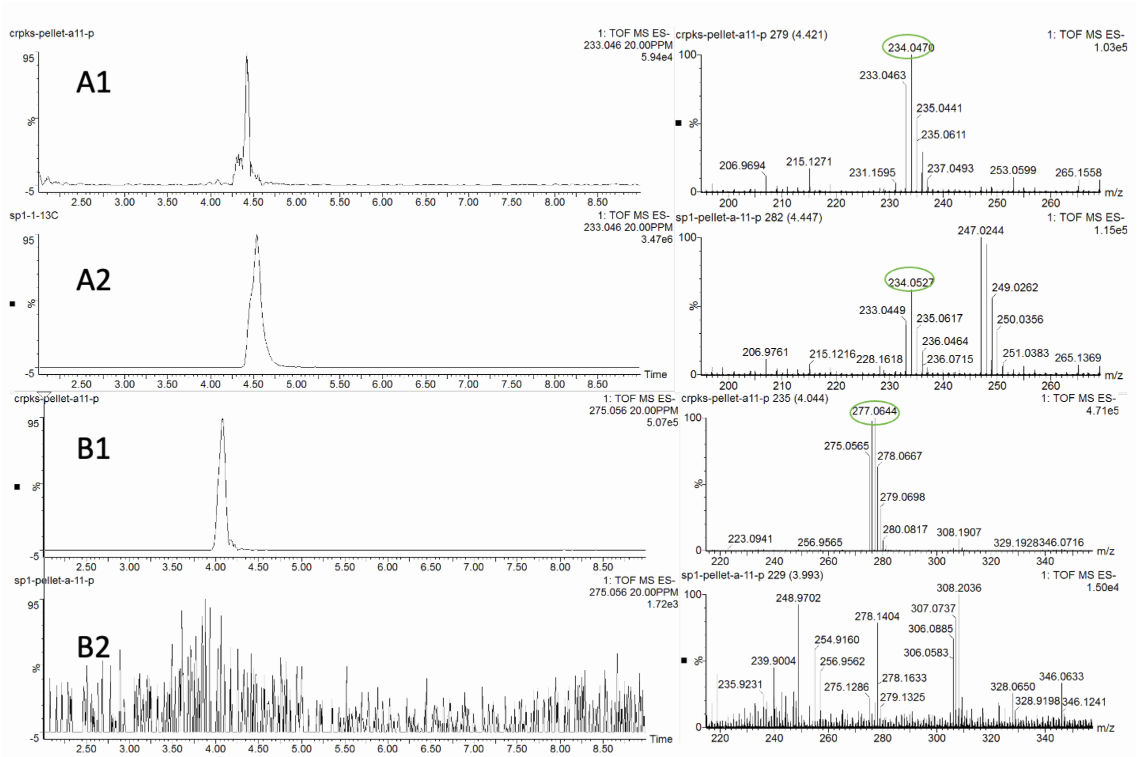

Figure S22: Cell pellet experiments using CrPKS and SpPks1, elated to Figure 5 and STAR Methods. A) ATHN (**10**) was detected in both (A1) CrPKS and (A2) SpPks1 enzymatic reactions. B) YWA1 (**13**) was detected in (B1) CrPKS but not (B2) SpPks1 enzymatic reactions. ^{13}C-acetate incorporated molecular ions are shown at right.

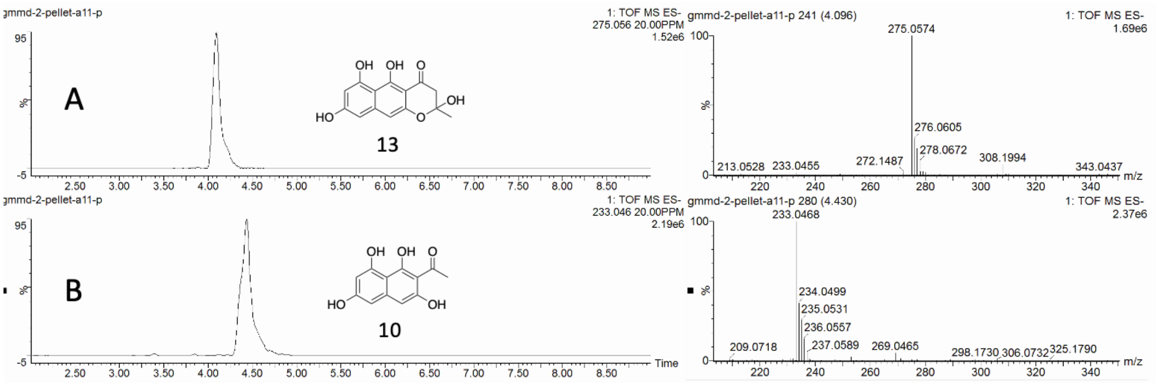

Figure S23: CrPKS-GMMD pellet experiments, related to Figure 5 and STAR Methods. The yeast pellet containing CrPKS-GMMD incubated with acetate solution. Extracted ion chromatograms of A) YWA1 (**13**) and B) ATHN (**10**). The mass spectrum at right shows ^{13}C-acetate incorporated.

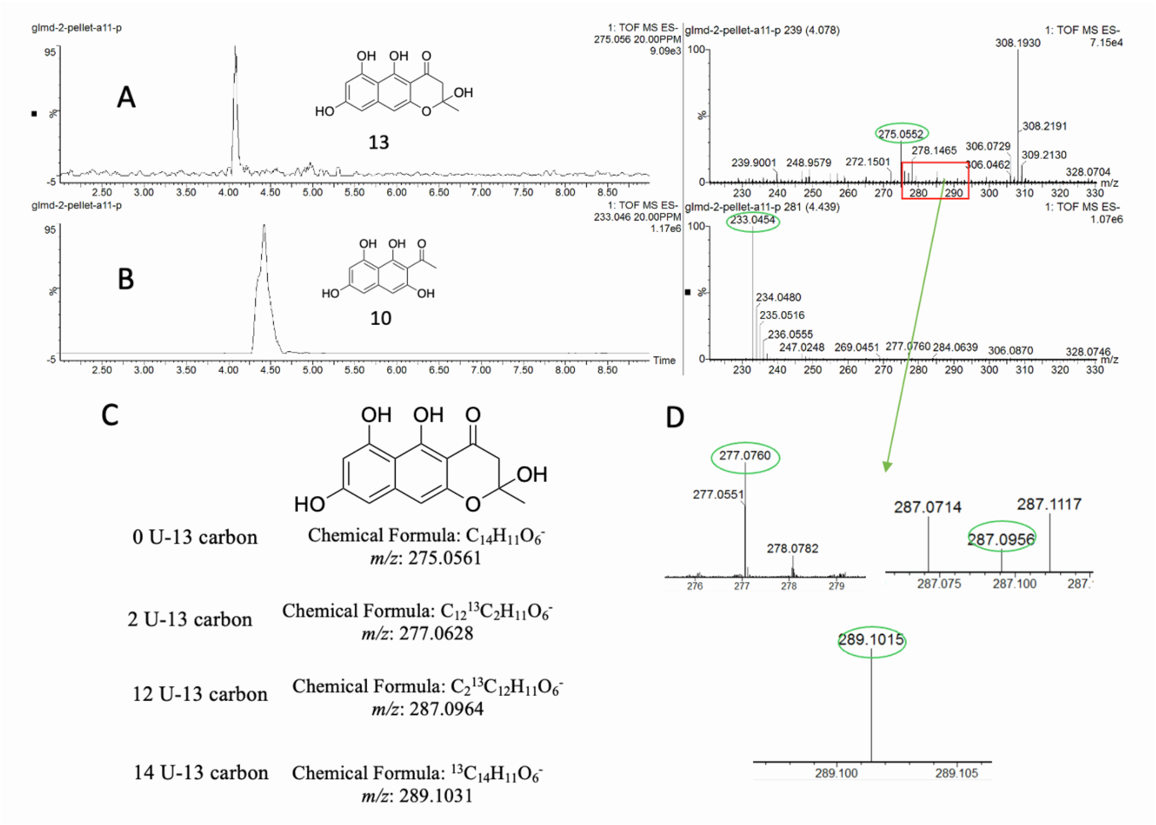

Figure S24: YWA1 (13) was detected in pellet experiments using SpPks1-GLMD, related to Figure 5 and STAR Methods. The yeast pellet containing SpPks1-GLMD incubated with acetate solution. Extracted ion chromatograms of A) YWA1 (**13**) and B) ATHN (**10**). C) and D) Representative [U-^{13}C]-malonate incorporation, showing the calculated *m/z* and mass spectra.

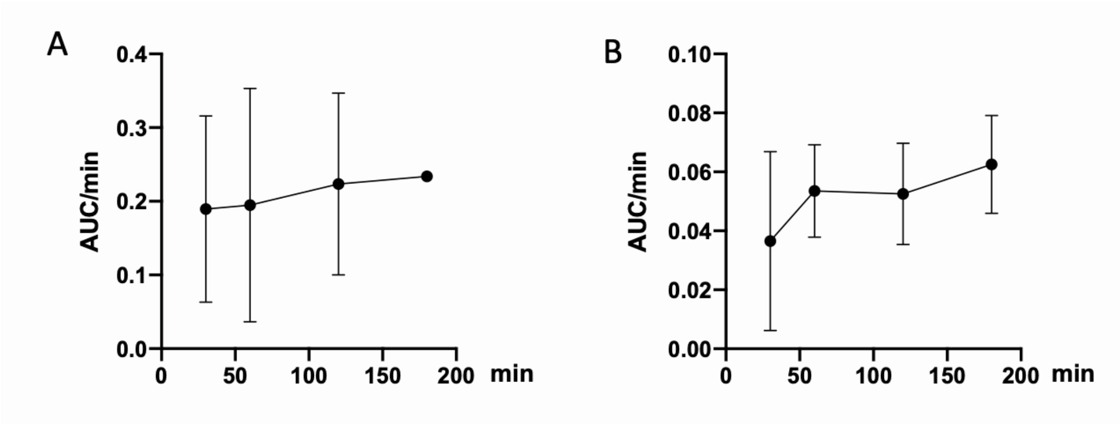

Figure S25: Time course experiment used to determine conditions for kinetics, related to Figure 5 and STAR Methods. A) CrPKS and B) CrPKS-GMMD reaction with butyryl-CoA (2 mM) and malonyl-CoA (2 mM), showing area under the curve (AUC) for compound **13**.

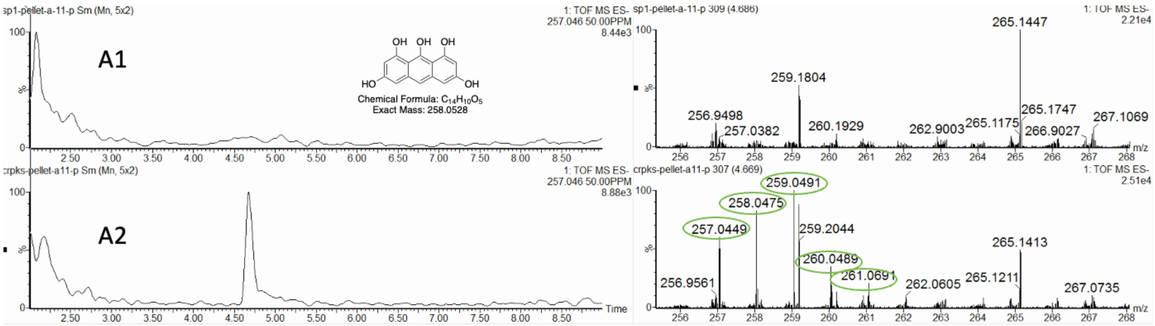

Figure S26: Putative anthraquinone precursor in yeast pellet experiment using CrPKS, related to Figure 6). (A1) Using a yeast pellet of SpPks1 as control, putative anthraquinone was detected in (A2) CrPKS.^{13}C-acetate-derived peaks are circled.

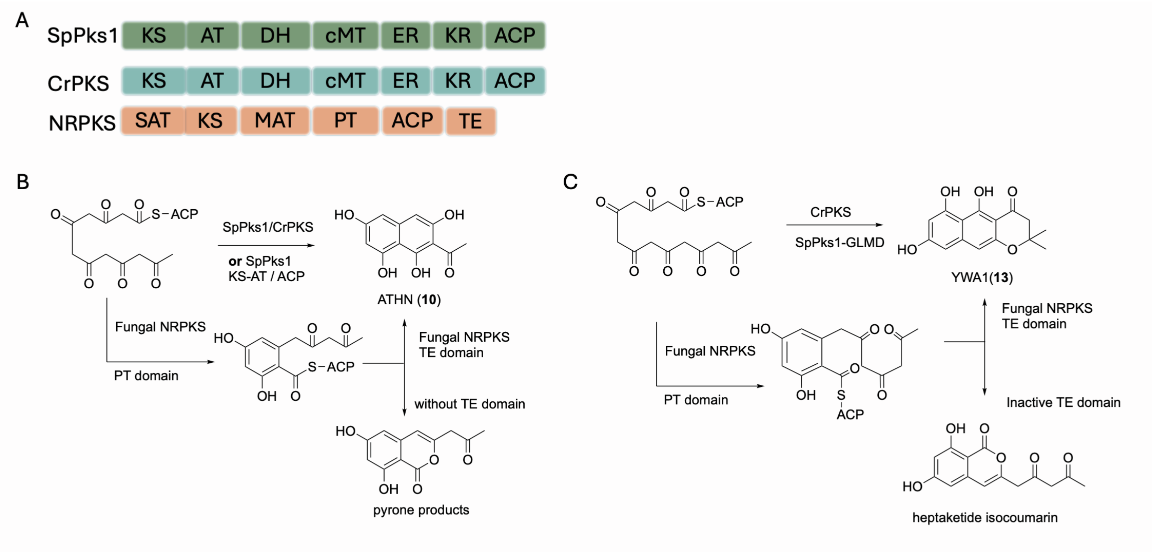

Figure S27: Echinoderm PKSs have a different biosynthetic mechanism than fungal NRPKS that synthesize the same compounds, related to Figure 6. A) Domain structure of echinoderm PKSs and NRPKS. B) Biosynthetic pathway to ATHN (**10**). C) Biosynthetic pathway to YWA1 (**13**).[11,45]

Table S1: Primers used in this study, related to Figure 5.

Primer	Sequence
Cr-GMMD-f	GCATCGGTATGATGGACTATC
Cr-GMMD-r	CTACAAATACACCACACG
Sp-GLMD-f	GTGATCCATCAAACCAATACCGAC
Sp-GLMD-r	GCCATCCAGCTAGTCG
